# Supplementary material for: Determinants of voluntary disclosure: An empirical analysis of financial, market, and organizational factors
Source: PLoS One. 2025 Jun 4;20(6):e0324625. doi: 10.1371/journal.pone.0324625 (PMC12136342; doi:10.1371/journal.pone.0324625)

**S3(Supporting Information 3): Overall Evaluation of the Logistic Regression Model**

**Table 3-1: Accuracy**

| Metric | Value |
| --- | --- |
| Accuracy | 0.8918 |

- The model's accuracy is 89.18%, indicating a high overall precision.

**Table 3-2: Confusion Matrix**

| Prediction/Actual | Non-voluntary Disclosure (VR=0) | Voluntary Disclosure (VR=1) |
| --- | --- | --- |
| Predicted as VR=0 | 33,826 | 1,486 |
| Predicted as VR=1 | 3,326 | 5,840 |

- There are 33,826 correct predictions for non-voluntary disclosure (VR=0) and 1,486 incorrect predictions.
- There are 5,840 correct predictions for voluntary disclosure (VR=1) and 3,326 incorrect predictions.

**Table 3-3: Classification Report**

| Class | Precision | Recall | F1-Score | Support |
| --- | --- | --- | --- | --- |
| VR=0 | 0.91 | 0.96 | 0.93 | 35,312 |
| VR=1 | 0.80 | 0.64 | 0.71 | 9,166 |
| Overall | 0.89 | 0.89 | 0.89 | 44,478 |
| Macro Avg | 0.85 | 0.80 | 0.82 | - |
| Weighted Avg | 0.89 | 0.89 | 0.89 | - |

- For VR=0, precision is 0.91, recall is 0.96, and the F1-score is 0.93, all of which are very high.
- For VR=1, precision is 0.80, recall is 0.64, and the F1-score is 0.71.

**Table 3-4: AUC (Area Under the ROC Curve)**

| **Metric** | **Value** |
| --- | --- |
| **AUC** | **0.9449** |

- The model's AUC is 0.9449, demonstrating a very high ability to distinguish between voluntary disclosure (VR=1) and non-voluntary disclosure (VR=0).


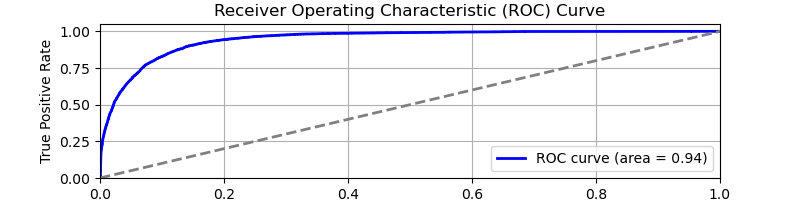

Supplement: S3 File — Results of the logistic regression model evaluation, including accuracy, confusion matrix, classification report, and area under the ROC curve (AUC). (DOCX) [file pone.0324625.s003.docx]
