# Supplementary material for: Determinants of voluntary disclosure: An empirical analysis of financial, market, and organizational factors
Source: PLoS One. 2025 Jun 4;20(6):e0324625. doi: 10.1371/journal.pone.0324625 (PMC12136342; doi:10.1371/journal.pone.0324625)
Supplement: Attachment — Submitted filename: PONE-D-25-00849_reviewer.pdf [file pone.0324625.s006.pdf]

Determinants of voluntary disclosure: An empirical analysis of financial, market, and organizational factors
PONE-D-25-00849

This manuscript examines the impact of financial indicators, corporate characteristics, stock exchange listings, industry classifications, and shareholder composition on voluntary disclosure. Using data from 5,915 listed firms in Japan from 2009 to 2024, the study finds that ISO certification, company size, and listing on a prime market are positively associated with higher voluntary disclosure. Furthermore, firms listed on foreign markets tend to actively disclose their non-financial reports. In sectoral analysis, the manufacturing and information technology sectors exhibit greater voluntary disclosure compared to the agriculture, forestry, fishing, and mining sectors. Additionally, institutional investors play a significant role in monitoring firms' disclosure practices. My general impression is that the topic is interesting. However, I have some concerns:

Major Issues:

1. This abstract is engaging! However, consider reorganizing it using the 3P+1T structure: Purpose, Procedure (Methods), Primary Findings, and Theory. Try to reduce it to 200 words.
2. For each hypothesis discussion, please provide supporting literature to strengthen your analysis.

Minor Issues:

3. In line 386, what is the explanatory variable **XXX**? Should the authors provide further clarification, or is this a typo?
4. The article's format and references appear inconsistent. Please double-check the formatting.
