# Supplementary material for: Determinants of voluntary disclosure: An empirical analysis of financial, market, and organizational factors
Source: PLoS One. 2025 Jun 4;20(6):e0324625. doi: 10.1371/journal.pone.0324625 (PMC12136342; doi:10.1371/journal.pone.0324625)
Supplement: Attachment — Submitted filename: PONE-D-25-00849_reviewer.docx [file pone.0324625.s007.docx]

**PLOS ONE**

**Determinants of voluntary disclosure: An empirical analysis of financial, market, and organizational factors**

--Manuscript Draft--

| **Manuscript Number:** | PONE-D-25-00849 |
| --- | --- |
| **Article Type:** | Research Article |
| **Full Title:** | Determinants of voluntary disclosure: An empirical analysis of financial, market, and organizational factors |
| **Short Title:** | Determinants of voluntary disclosure in Japan |
| **Corresponding Author:** | Yuichiro Nakai, M.D  Tsukuba Daigaku - Tokyo Campus Bunkyo Kosha Tokyo, JAPAN |
| **Keywords:** | voluntary disclosure, foreign listing, ISO certification, shareholder composition, logistic regression analysis, Japanese Companies |
| **Abstract:** | The purpose of this study is to systematically examine the determinants of voluntary disclosure by listed companies in Japan. Voluntary disclosure refers to the disclosure of information that is not legally required, such as CSR reports and ESG reports. In Japan, the disclosure of such non-financial information is not legally required and is left to the discretion of individual companies. As a result, it provides an ideal case study for analyzing the factors influencing voluntary disclosure behavior. This study uses quarterly data from 5,915 listed companies in Japan from 2009 to 2024 to examine the impact of five key factors on voluntary disclosure using binary logistic regression analysis: financial indicators, company characteristics, stock exchange, industry classification, and shareholder composition. The main findings of this study are summarized as follows: 1) ISO certification (ISO 14001 and ISO 45001) significantly promotes voluntary disclosure. 2) Company size and listing on a prime market are positively associated with voluntary disclosure. 3) Listing in foreign markets tends to suppress voluntary disclosure due to differences in disclosure standards and market environments. 4) The manufacturing and information technology sectors are proactive in disclosure, while the agriculture, forestry fishing, and mining sectors are more reluctant. 5) Shareholder composition influences disclosure behavior, with financial institution shareholders promoting disclosure and other corporate shareholders suppressing it. Based on the unique voluntary disclosure environment in Japan, this study provides new insights into the diversity of voluntary disclosure behavior. It offers valuable suggestions for increasing corporate transparency, pursuing sustainability, and designing effective disclosure strategies and policies. |
| **Order of Authors:** | Yuichiro Nakai, M.D |
|  | Mitsuo Yoshida |
| **Additional Information:** |  |
| **Question** | **Response** |
| **Financial Disclosure**  Enter a financial disclosure statement that describes the sources of funding for the work included in this submission. Review the [submission guidelines](http://journals.plos.org/plosone/s/submission-guidelines#loc-financial-disclosure-statement) for detailed requirements. View published research articles from [*PLOS ONE*](https://journals.plos.org/plosone/) for specific examples.  This statement is required for submission | The author(s) received no specific funding for this work. |

| and **will appear in the published article** if the submission is accepted. Please make sure it is accurate. |  |
| --- | --- |
| **Funded studies**  Enter a statement with the following details:   - Initials of the authors who received each award - Grant numbers awarded to each author - The full name of each funder - URL of each funder website - Did the sponsors or funders play any role in the study design, data collection and analysis, decision to publish, or preparation of the manuscript? |  |
| Did you receive funding for this work? |  |
| **Competing Interests**  Use the instructions below to enter a competing interest statement for this submission. On behalf of all authors, disclose any [competing interests](http://journals.plos.org/plosone/s/competing-interests) that could be perceived to bias this work—acknowledging all financial support and any other relevant financial or non- financial competing interests.  This statement is required for submission and **will appear in the published article** if the submission is accepted. Please make sure it is accurate and that any funding sources listed in your Funding Information later in the submission form are also declared in your Financial Disclosure statement.  View published research articles from  [*PLOS ONE*](http://journals.plos.org/plosone/) for specific examples. | The authors have declared that no competing interests exist. |

| **NO authors have competing interests**  Enter: *The authors have declared that no competing interests exist.*  **Authors with competing interests**  Enter competing interest details beginning with this statement:  *I have read the journal's policy and the authors of this manuscript have the following competing interests: [insert competing interests here]* |  |
| --- | --- |
| * typeset |  |
| **Ethics Statement**  Enter an ethics statement for this submission. This statement is required if the study involved:   - Human participants - Human specimens or tissue - Vertebrate animals or cephalopods - Vertebrate embryos or tissues - Field research   Write "N/A" if the submission does not require an ethics statement.  General guidance is provided below. Consult the [submission guidelines](http://journals.plos.org/plosone/s/submission-guidelines#loc-guidelines-for-specific-study-types) for detailed instructions. **Make sure that all information entered here is included in the Methods section of the manuscript.** | N/A |

| **Format for specific study types**  **Human Subject Research (involving human participants and/or tissue)**   - Give the name of the institutional review board or ethics committee that approved the study - Include the approval number and/or a statement indicating approval of this research - Indicate the form of consent obtained (written/oral) or the reason that consent was not obtained (e.g. the data were analyzed anonymously)   **Animal Research (involving vertebrate animals, embryos or tissues)**   - Provide the name of the Institutional Animal Care and Use Committee (IACUC) or other relevant ethics board that reviewed the study protocol, and indicate whether they approved this research or granted a formal waiver of ethical approval - Include an approval number if one was obtained - If the study involved *non-human primates*, add *additional details* about animal welfare and steps taken to ameliorate suffering - If anesthesia, euthanasia, or any kind of animal sacrifice is part of the study, include briefly which substances and/or methods were applied   **Field Research**  Include the following details if this study involves the collection of plant, animal, or other materials from a natural setting:   - Field permit number - Name of the institution or relevant body that granted permission |  |
| --- | --- |
|  |  |
| **Data Availability**  Authors are required to make all data underlying the findings described fully available, without restriction, and from the time of publication. PLOS allows rare exceptions to address legal and ethical concerns. See the [PLOS Data Policy](http://journals.plos.org/plosone/s/data-availability) and [FAQ](http://journals.plos.org/plosone/s/data-availability#loc-faqs-for-data-policy) for detailed information. | Yes - all data are fully available without restriction |

| A Data Availability Statement describing where the data can be found is required at submission. Your answers to this question constitute the Data Availability Statement and **will be published in the article**, if accepted. |  |
| --- | --- |
| **Important:** Stating ‘data available on request from the author’ is not sufficient. If your data are only available upon request, select ‘No’ for the first question and explain your exceptional situation in the text box. |  |
| Do the authors confirm that all data underlying the findings described in their manuscript are fully available without restriction? |  |
| **Describe where the data may be found in full sentences. If you are copying our sample text, replace any instances of XXX with the appropriate details.**   - If the data are **held or will be held in a public repository**, include URLs, accession numbers or DOIs. If this information will only be available after acceptance, indicate this by ticking the box below. For example: *All XXX files are available from the XXX database (accession number(s) XXX, XXX.*). - If the data are all contained **within the manuscript and/or Supporting Information files**, enter the following: *All relevant data are within the manuscript and its Supporting Information files.* - If neither of these applies but you are able to provide **details of access elsewhere**, with or without limitations, please do so. For example:   *Data cannot be shared publicly because of [XXX]. Data are available from the XXX Institutional Data Access / Ethics Committee (contact via XXX) for researchers who meet the criteria for access to confidential data.*  *The data underlying the results presented in the study are available from (include the name of the third party* | All relevant data are within the manuscript and its Supporting Information files. |

| *and contact information or URL).*   - This text is appropriate if the data are owned by a third party and authors do not have permission to share the data.   * typeset |  |
| --- | --- |
| Additional data availability information: |  |

<https://www2.cloud.editorialmanager.com/pone/download.aspx?id=38869333&guid=faa61394-871d-410f-98a8-736d5591af02&scheme=1>Manuscript	[Click here to](https://www2.cloud.editorialmanager.com/pone/download.aspx?id=38869333&guid=faa61394-871d-410f-98a8-736d5591af02&scheme=1)

[access/download;Manuscript;Manuscript_2025_0107_1147.do](https://www2.cloud.editorialmanager.com/pone/download.aspx?id=38869333&guid=faa61394-871d-410f-98a8-736d5591af02&scheme=1)

1

2

3

4 *Determinants of voluntary disclosure: An empirical analysis of financial, market, and organizational factors*
5 *Determinants of voluntary disclosure in Japan*

6

7	Yuichiro Nakai^1^*, Mitsuo Yoshida^2^

8

9 ^1^ Degree Programs in Business Sciences, University of Tsukuba, Tokyo, Japan
10 ^2^ Institute of Business Sciences, University of Tsukuba, Tokyo, Japan

11

12 * Corresponding author
13 Email: [s2440411@u.tsukuba.ac.jp](mailto:s2440411@u.tsukuba.ac.jp) (Y.N)

14

15	**Abstract**

16 The purpose of this study is to systematically examine the determinants of voluntary disclosure by listed
17 companies in Japan. Voluntary disclosure refers to the disclosure of information that is not legally required, such as
18 CSR reports and ESG reports. In Japan, the disclosure of such non-financial information is not legally required and
19 is left to the discretion of individual companies. As a result, it provides an ideal case study for analyzing the factors
20 influencing voluntary disclosure behavior. This study uses quarterly data from 5,915 listed companies in Japan from
21 2009 to 2024 to examine the impact of five key factors on voluntary disclosure using binary logistic regression
22 analysis: financial indicators, company characteristics, stock exchange, industry classification, and shareholder
23 composition. The main findings of this study are summarized as follows: 1) ISO certification (ISO 14001 and ISO
24 45001) significantly promotes voluntary disclosure. 2) Company size and listing on a prime market are positively
25 associated with voluntary disclosure. 3) Listing in foreign markets tends to suppress voluntary disclosure due to
26 differences in disclosure standards and market environments. 4) The manufacturing and information technology
27 sectors are proactive in disclosure, while the agriculture, forestry fishing, and mining sectors are more reluctant. 5)
28 Shareholder composition influences disclosure behavior, with financial institution shareholders promoting
29 disclosure and other corporate shareholders suppressing it. Based on the unique voluntary disclosure environment
30 in Japan, this study provides new insights into the diversity of voluntary disclosure behavior. It offers valuable
31 suggestions for increasing corporate transparency, pursuing sustainability, and designing effective disclosure
32 strategies and policies.

33

34 **Keywords:** voluntary disclosure, foreign listing, ISO certification, shareholder composition, logistic regression
35 analysis, Japanese Companies

36

# 37 1. Introduction

38 Why do companies choose to disclose information they are not legally obligated to disclose?” What is behind
39 the voluntary CSR and ESG reporting by many Japanese companies, despite the lack of legal obligation?” Due to
40 the changing nature of motivation and scope of disclosure over time, there are many studies on disclosure, and it
41 is essential to examine the current disclosure issues. Therefore, we examine this issue by organizing corporate
42 disclosure into two types: statutory and voluntary shown in Table 1.

##### 43 Table 1 Classification of corporate disclosure and the position of voluntary disclosure in this report

| Disclosure (major category) | Disclosure (Medium Category) | Classification. | Example | Contents | nature |
| --- | --- | --- | --- | --- | --- |
| Statutory disclosure (broadly defined) | Statutory disclosure (narrowly defined) | Legal, etc. (hard law) | Annual Securities Report, Financial Statements | Focus on financial information | IR |
|  | Statutory disclosure (Other) | Market rules, etc. (soft law) | Corporate Governance Report | Financial and non-financial information |  |
| Voluntary disclosure (broadly defined) | Voluntary disclosure (supplemental to statutory disclosure) | Public Relations Activities for the Press | Press Release | Financial and non-financial information,  Product information, etc. | IR-  oriented |
|  | （ Voluntary Disclosure (in this paper) | corporate activities | CSR Reporting, ESG Reporting, SDGs Reporting, Integrated Reporting, Sustainability Reporting |  |  |
|  | Voluntary Disclosure (Other) | propaganda activities | Commercials, etc. | Product information, etc. | PR |

44 Statutory disclosures are disclosures of financial information required by law or regulation and include annual
45 reports, financial statements, and corporate governance reports. These are intended to ensure transparency to investors
46 and regulators. In recent years, non-financial information has been increasingly recognized as an increasingly
47 important part of statutory disclosure [1,2], but its scope remains limited. On the other hand, companies may choose
48 to use voluntary disclosures to supplement statutory disclosures. Voluntary disclosures are additional information not
49 required by Generally Accepted Accounting Principles (GAAP) or specific regulations [3] and include CSR reporting,
50 ESG reporting, and integrated reporting, as companies voluntarily provide financial and non-financial information
51 not required by statutory disclosure. Voluntary disclosure is becoming increasingly important in today's capital
52 markets because it helps companies better identify the social responsibilities [4] they assume and activities that create
53 value for investors and reduce information asymmetries between shareholders and management [5]. While voluntary
54 disclosure is important in building investor and stakeholder trust, it also entails a conflict between transparency and
55 maintaining competitive advantage, due to the need to avoid providing too much information. In addition, it is
56 difficult to directly measure a company's emotional and internal decision-making processes, and this poses a
57 challenge when understanding corporate behavior from the outside. Therefore, externally available objective data
58 must be used to infer corporate decision-making.
59 However, there are few studies that have conducted exploratory and comprehensive analyses of the factors that
60 encourage companies to actively disclose information and the factors that discourage them from doing so.
61 Japan is unique in this regard. Companies there are not legally required to disclose non-financial information, such
62 as CSR and ESG reporting. Instead, they are free to make their own choices. This allows for interesting comparisons
63 between companies that disclose such information and those that do not. This unique situation makes Japan an
64 important case in international comparative studies. It is also very important to analyze how voluntary disclosure is
65 reflected in corporate strategy. We can do this by comparing it with mandatory disclosure standards in other countries,
66 such as South Africa and the EU. The purpose of this study is to explore and clarify the decision-making factors that
67 influence Japanese Companies' choice of voluntary disclosure. Specifically, we systematically examine how these
68 factors affect voluntary disclosure from five perspectives: financial indicators, Company characteristics, listed market,
69 industry classification, and shareholder composition.
70 The period analyzed in this study is the quarterly data from March 2009 to March 2024, covering a total of 5,915
71 Japanese listed companies during this period. Based on these data, we used logistic regression analysis to examine
72 the effects of financial indicators, Company characteristics, listing market, industry classification, and shareholder
73 composition.
74 As a result, the following key findings were obtained
75 1. academic originality and novelty
76 ⚫ Foreign Market Listings Suppress Voluntary Disclosure: While conventional theory associates foreign
77 market listings with increased transparency and disclosure, this study finds the opposite.
78 ⚫ Companies with higher P/E ratios are less likely to voluntarily disclose: The finding that companies with
79 higher growth expectations tend to refrain from disclosing for competitive reasons is a new finding that
80 contradicts conventional theory.
81 ⚫ ISO certification promotes disclosure: ISO 14001 and ISO 45001 certifications were found to function as
82 factors that actively promote voluntary disclosure.
83 2. consistency with research objectives
84 • Company size (total assets and sales) promotes voluntary disclosure: The tendency for larger companies to
85 actively engage in voluntary disclosure was clearly confirmed.
86 • Increased voluntary disclosure activity in prime markets: The results show that differences in market
87 segmentation have a strong influence on Companies' voluntary disclosure behavior.
88 • Trends by industry category: The manufacturing and information and communications industries were
89 highly proactive, while the agriculture, forestry, fisheries, and mining industries showed a markedly restrained
90 trend.
91 3. practical and policy contributions
92 • Demand for transparency in the prime market: The increased activity of voluntary disclosure in the prime
93 market reflects the demand for transparency from investors and has policy implications.
94 • Differences by shareholder composition: Voluntary disclosure is promoted when financial institutions are
95 the major shareholders, while it is suppressed when there are many other corporate shareholders.
96 • Practical impact of ISO certification: Environmental and occupational safety initiatives are specifically
97 linked to the company's information disclosure strategy.

98

99 The novelty of this study lies in its comprehensive analysis of corporate decision making, taking advantage of
100 Japan's unique voluntary disclosure environment. In particular, the finding that listing on a foreign market suppresses
101 voluntary disclosure, contrary to existing theory, is a unique result of this study. These results provide new insights
102 into the voluntary disclosure behavior of Japanese companies, deepen our understanding of corporate strategy and
103 fulfillment of social responsibility in a non-regulatory environment, and provide practical implications for
104 international disclosure standards and sustainability strategies.

105

# 106 2. Literature review

107 In this chapter, we organize disclosure theory and prior research to develop a theoretical framework for identifying
108 factors that influence voluntary disclosure by Japanese Companies. The literature review has two aspects: prior
109 knowledge and prior research. Since the purpose of this paper is to provide a comprehensive, exploratory analysis of
110 the factors affecting voluntary disclosure, the selected literature provides a basis for developing theories and
111 hypotheses supporting voluntary disclosure from the following perspectives. First, we introduce some specific
112 theories, country systems, and the Japanese situation that support our study. Second, we introduce previous studies
113 relevant to our study and identify in detail the theoretical and empirical bases for each of our hypotheses.

114

## 115 2.1 Background knowledge

### 116 2.1.1 Disclosure Theory and Prior Research

117 Based on agent theory, companies are encouraged to disclose information to eliminate information asymmetries
118 and increase transparency [6]. However, excessive disclosure also carries the risk of losing a competitive advantage,
119 so careful judgment is required [7,8]. There is much discussion about how corporate disclosure affects strategy and
120 its intentions. Some have noted that "disclosure is one of the most important decisions in business" and that it affects
121 corporate strategy, stakeholders, and even market valuation [9]. In other words, it is important to note that information
122 is not simply a matter of disclosure. There is also debate about the roles and interactions between voluntary and
123 statutory disclosure. For example, while there is little coordination between investor relations (IR) and general public
124 relations (PR) activities, it has been pointed out that an appropriate combination of voluntary and statutory disclosure
125 may create synergy [10]. Some suggest that integrated reporting (IR), which deals with financial and non-financial
126 information, combines traditional financial accounting with sustainability and corporate governance issues to
127 enhance decision usefulness and play a key role in business management and stakeholder relations management [11].
128 These studies suggest that corporate disclosure is heavily influenced by strategy, business environment, and even
129 market expectations. Initiative-taking disclosure increases transparency and strengthens trust with investors and
130 regulators. However, excessive disclosure also carries the risk of losing a competitive advantage, so prudent and
131 strategic decisions must be made.

132

### 133 2.1.2 Comparison of International Disclosure Systems

134 Currently, due to differences in the legal and other systems of various countries, international differences in the
135 range of information disclosures are recognized. For example, South Africa has required companies listed on the
136 Johannesburg Stock Exchange to submit integrated reports since 2010 (King Code Chapter 9). In France, CSR
137 reporting has been mandatory since 2001 [12], and the EU passed a law requiring the disclosure of non-financial
138 information in 2014 [13]. In Italy, large companies with more than five hundred employees are required to disclose
139 SDG-related information from 2017 [14], among other differences. In Europe, the Value Reporting Foundation (VRF),
140 established by the merger of the International Integrated Reporting Council (IIRC) and the Sustainability Accounting
141 Standards Board (SASB), and the IFRS Foundation (https://[www.ifrs.org/news-and-events/news/2022/08/ifrs](http://www.ifrs.org/news-and-events/news/2022/08/ifrs) -
142 foundation-completes-consolidation-with-value-reporting-foundation/), and international standards for ESG
143 information have been developed (https://[www.ifrs.org/news-and-events/news/](http://www.ifrs.org/news-and-events/news/) 2022/08/ifrs-foundation-completes-
144 consolidation-with-value-reporting-foundation/). This consolidation will improve the consistency and comparability
145 of information and meet the requirements of international investors.
146 According to the author's tabulations, as shown in Fig 1, it is recognized that Japanese companies have a history
147 of preparing voluntary reports under such names as "Annual Report," "CSR Report," and "ESG Report" to meet
148 international requirements as appropriate. Recently, there has been a noticeable trend to discontinue "CSR Reports"
149 and "ESG Reports" and to integrate information into "Integrated Reports" and "Company Reports.”

150

151 Fig1. Trends in Voluntary Disclosure Report Names for Japanese Listed Companies 2008-2023.
152 This figure highlights the significant increase in the number of Integrated Reports and Company Name Reports, as well as the notable decrease in the
153 number of Annual Reports issued by Japanese listed companies from 2008 to 2023.

154

155 In Japan, there is no legal obligation for companies to disclose environmental and social responsibility information,
156 and companies are free to choose whether or not to do so on a voluntary basis. CSR and ESG reporting are the most
157 common forms of voluntary disclosure by Japanese companies, and most of them are provided in the form of
158 integrated reports and company reports. These reports are unique in that they conform to international standards such
159 as the Global Reporting Initiative (GRI) and International Financial Reporting Standards (IFRS), while at the same
160 time reflecting regional characteristics and cultural backgrounds. The voluntary nature of the standard is a feature of
161 the environment. This environment of voluntary nature makes Japan an ideal case for exploring the factors and
162 motivations that influence voluntary disclosure behavior.

163

164 The number of listed companies and the number of voluntary disclosers in Japan from 2009 to 2024, Fig2 shows
165 that the number of voluntary disclosers is increasing, but not all companies are actively engaged in voluntary
166 disclosure. In this regard, identifying the factors that cause companies to engage in voluntary disclosure is the subject
167 of this study.

168

169 Fig2. Trends in Listed and Voluntary Disclosure Companies in Japan (2009-2024)
170 This figure shows the trend in the total number of listed companies in Japan and the number of companies engaging in voluntary disclosure. The right
171 axis represents the percentage of companies practicing voluntary disclosure, displaying a consistent upward trend over the period.

172

173 Voluntary disclosure is part of corporate strategy and a means of demonstrating corporate transparency and social
174 responsibility to investors and stakeholders. However, it remains unclear which factors influence voluntary disclosure.
175 While there has been research on the impact of the combination of statutory and voluntary disclosure on corporate
176 valuation, there is limited knowledge on how corporate characteristics and market differences are reflected in
177 voluntary disclosure. In addition, since decisions regarding voluntary disclosure are made internally within a
178 company, it is difficult to study the process from the outside. Nevertheless, it is clear that Companies make voluntary
179 disclosure decisions in consideration of the business environment. The purpose of this study is to analyze the
180 characteristics of companies that disclose voluntarily and those that do not, based on publicly available information
181 such as corporate websites and financial reports, and to identify the factors that contribute to voluntary disclosure.

182

## 183 2.2 Previous Studies and Research Hypotheses

184 This study defines voluntary disclosure as additional information that is not required by Generally Accepted
185 Accounting Principles (GAAP) or specific regulations but is voluntarily disclosed by companies. Specifically, CSR
186 reporting, ESG reporting, SDGs reporting, integrated reporting, and sustainability reporting prepared by Japanese
187 listed companies fall into this category, and while these reports are not legally required, they are positioned as
188 important means of demonstrating corporate strategy and social responsibility. The purpose of this study is to
189 elucidate the factors that lead Japanese listed companies to choose to disclose these voluntary disclosures. Specifically,
190 we aim to identify the factors that influence voluntary corporate disclosure behavior from the five perspectives of
191 financial indicators, corporate characteristics, listed market, industry classification, and shareholder composition, in
192 order to gain a comprehensive understanding of how companies fulfill their transparency and social responsibility.
193 Furthermore, based on these factors, the following hypotheses will be formulated
194 The following hypotheses were developed for this study in order to identify the factors that influence voluntary
195 disclosure by Companies. These hypotheses examine how financial indicators, Company characteristics, listing
196 market, industry classification, and shareholder composition affect voluntary disclosure and are in a form that allows
197 for testing whether the results are positive, negative, or irrelevant.

198

### 199 2.2.1 Hypothesis 1: The impact of financial measures on voluntary

200 **disclosure**
201 A number of prior studies have suggested that financial indicators affect voluntary disclosure by Companies. It has
202 been suggested that Companies with higher market capitalization reduce the cost of capital by disclosing integrated
203 information , suggesting that stock market capitalization affects voluntary disclosure [15]. In addition to reports
204 confirming that Companies with larger total assets disclose more information [16], there are also indications that
205 larger Companies tend to report more detail and expand the scope of disclosure than smaller Companies [17].
206 Therefore, capitalization and the number of shares issued may also affect voluntary disclosure. Sales (Sales) and
207 Operating Profit (Operating Profit), as indicators of company size, are expected to positively influence voluntary
208 disclosure [18] , but it has been pointed out that highly competitive companies, especially those with high profit
209 margins, may not disclose information on sales and costs [19] , so the impact may be both positive and It could occur
210 passively. While dividend per share (DPS) indicates that dividends tend to increase in a transparent environment ,
211 DPS is expected to have an impact on voluntary disclosure [20], as there are indications that mandatory CSR
212 disclosure will significantly reduce the amount of dividends paid by companies [21] . Finally, the price-to-book ratio
213 (PBR) and price-to-earnings ratio (PER), which are ratios of the financial indicators presented above, are thought to
214 influence disclosure behavior as indicators of a company's market valuation. A company with a high PER indicates
215 high growth expectations, which may discourage voluntary disclosure, while a company with a low PER indicates a
216 company with high growth expectations, which may discourage voluntary disclosure, P/B ratio reflects the value of
217 a company's assets and is considered to be a factor that promotes disclosure.
218 Based on these studies, we believe that clarifying the impact of financial indicators on voluntary disclosure by
219 Companies will help us understand how Companies strategically disclose information. Therefore, hypothesis H1
220 expects a meaningful relationship to exist between these financial indicators and voluntary disclosure reporting.

221

##### 222 H1: Financial indicators influence the degree of voluntary disclosure.

223

### 224 2.2.2 Hypothesis 2: The impact of corporate characteristics on voluntary

225 **disclosure**
226 A number of prior studies have also suggested that corporate characteristics influence corporate voluntary
227 disclosure. In addition to the suggestion that companies with a larger number of employees tend to disclose
228 information about employee training more proactively [22] , there are also indications that larger companies are more
229 likely to disclose social information [23] , suggesting that the number of employees has a positive impact on voluntary
230 disclosure. Regarding corporate age, several studies [24–26] found that corporate age does not have a significant
231 impact on voluntary disclosure, although more experienced Companies are likely to disclose more information to
232 enhance their reputation and image .It will be interesting to see if the same results are obtained in other countries, as
233 Japan has a large number of Companies with extremely high corporate age. On the other hand, it is reported that the
234 number of years listed has no significant effect on disclosure in Indonesia [27] , and it is possible that the length of
235 listing also has a limited effect on voluntary disclosure. Regarding the adoption of IFRS (International Financial
236 Reporting Standards), there are indications that IFRS may correct information deficiencies in financial statements
237 and influence the disclosure behavior of companies [28]; there are indications that the adoption of IFRS will improve
238 the quality and comparability of financial statements and increase the profitability of companies [29]; and GAAP
239 (Generally The choice of Generally Accepted Accounting Principles (GAAP) is also expected to impact voluntary
240 disclosure. With regard to listing in foreign markets, it has been shown that companies choose foreign stock markets
241 based on signaling models and that the market environment may influence disclosure [30] . It has also been suggested
242 that companies listed in multiple countries tend to disclose more ESG data than companies listed only in their home
243 market in order to meet the demands of external capital markets [31]. There are also reports that corporate accounting
244 disclosures are affected by investor demand for information, location, size, and listing on foreign markets [32]. There
245 are also reports that listing on a foreign market affects disclosure behavior in response to the internationalization of
246 legal systems and markets [33], and these factors may affect voluntary disclosure.
247 In addition, there are indications that share repurchases may intentionally increase unwelcome news because they
248 lower the purchase price at which management purchases shares [34]. There is also a report that says that high-value
249 companies buy back their own shares without disclosing them, increasing the liquidity of stock transactions and
250 consequently reducing the equilibrium disclosure area [35], so we consider share buybacks as a key factor that affects
251 the voluntary disclosure behavior of Companies. Finally, with regard to ISO certification, while reporting that ISO
252 certification has no direct impact on carbon emissions disclosure, profitability and Company size have a positive
253 impact on disclosure [36] . There is also a suggestion that ISO 14001 certification has no significant impact on
254 greenhouse gas emissions disclosure [37] , but Japan ISO certification is an important Explanation variable to
255 understand the impact on voluntary disclosure behavior of Japanese companies.
256 Based on these studies, we believe that identifying the impact of Company characteristics on voluntary disclosure
257 will help us understand how Companies formulate their disclosure strategies. Therefore, under Hypothesis H2, we
258 expect a meaningful relationship to exist between corporate characteristics and voluntary disclosure.

259

##### 260 H2: Corporate characteristics determine the extent of voluntary disclosure.

261

### 262 2.2.3 Hypothesis 3: The impact of listing market factors on voluntary

263 **disclosure**
264 Numerous prior studies have provided insights into the impact of the characteristics of the market on which a
265 company is listed on its voluntary disclosure. It has been found that the more stringent the regulations of a listed
266 market, the more aggressive Companies tend to disclose information to increase transparency [38,39]. It has also
267 been suggested that the strictness of the regulatory environment has a direct impact on corporate disclosure
268 behavior, creating an incentive to disclose more information [40].	As for the trend of voluntary disclosure in
269 Japan, as shown in Fig 3, which shows the trend by listing market based on Japanese data from 2009 to 2024, the
270 majority of companies that engage in voluntary disclosure are listed on the prime market.

271

272 Fig 3. Trends of Voluntary Disclosure by Market Segment in Japan (2009-2024)
273 This figure shows the trends of voluntary disclosure by market segment in Japan for the period from 2009 to 2024, highlighting the significant increase
274 in the number of companies engaging in voluntary disclosure within the Prime market segment.

275

276 This suggests that the prime market's strict regulations and high transparency requirements from investors may have
277 a strong influence on companies' disclosure behavior. Companies listed on the standard, growth, and regional markets
278 are expected to be more reluctant to disclose information because they are less regulated than those on the prime
279 market.
280 Thus, it is important in this study to examine how listed markets affect voluntary disclosure by Companies. By
281 analyzing the impact of specific market segments (prime market, standard market, growth market, and local market)
282 on voluntary disclosure in Japan, we expect to clarify the role of market segments on corporate disclosure behavior.

283

##### 284 H3: The listing market determines the extent of voluntary disclosure.

285

### 286 2.2.4 Hypothesis 4: The impact of industry classification on voluntary

287 **disclosure**
288 Many studies have shown that industry-specific characteristics influence corporate voluntary disclosure, pointing
289 out that the regulatory and competitive environment of each industry influences the scope and content of disclosure
290 [41,42]. Some have reported that voluntary disclosure is more active in the manufacturing industry, which has a
291 higher propensity to pollute the environment [43] , suggesting that industry characteristics have a significant impact
292 on disclosure strategies.	Fig 4, which shows Japanese Companies voluntarily disclosing information from 2009
293 to 2024 by industry category (10 major categories), also shows that Manufacturing (+306) accounts for a large
294 share of the total, indicating that industry category is a key factor in information disclosure.

295

296 Fig 4. Trends of Voluntary Disclosure by Major Industry Categories in Japan (2009-2024)
297 This figure shows the trend of voluntary disclosure across major industries in Japan, highlighting a significant increase in the manufacturing sector, which
298 has the highest number of companies engaged in voluntary disclosure from 2009 to 2024. The legend indicates the increase in the number of companies
299 practicing voluntary disclosure from March 2009 to March 2024 for each industry.

300

301 Commercial (+101), Transport, Information and communications (+87), Finance and Insurance (+74), and
302 Services (+66) also show a certain upward trend, while Mining (+1), Fishery, Agriculture & Forestry (+2), and
303 Manufacturing (+3) are also on the rise. Mining (+1), Fishery, Agriculture & Forestry (+3), Electricity, Gas (+7),
304 Real Estate (+15), and Construction (+35) have shown minor change in response to voluntary disclosure. Since
305 these industries have different practices, it is important for this study to examine the impact of these differences.
306 In particular, it is expected to clarify the role of industry characteristics on disclosure strategies by analyzing how
307 industry classification affects voluntary disclosure in Japan.

308

##### 309 H4: Industry classification determines the extent of voluntary disclosure.

310

### 311 2.2.5 Hypothesis 5: The impact of shareholder composition on voluntary

312 **disclosure**
313 A number of previous studies have suggested the impact of shareholder composition on voluntary corporate
314 disclosure. As so-called institutional investors, companies with a higher percentage of ownership by financial
315 institutions (life insurance companies, non-life insurance companies, trust banks, ordinary banks, pension funds, etc.)
316 tend to disclose information more proactively in order to increase transparency, according to [44] . In addition,
317 companies with major shareholders promote disclosure along with the introduction of independent directors [45] .
318 Furthermore, it has been noted that sustainability-related disclosures have a positive and meaningful relationship with
319 foreign and institutional shareholder composition and board independence [46] . Thus, shareholder composition has
320 attracted attention as a factor that influences corporate disclosure behavior. In particular, companies in which financial
321 institutions are major shareholders may promote voluntary disclosure because of the strong external demand for
322 transparency. On the other hand, companies with many other corporations or individual shareholders are expected to
323 face less pressure to disclose information and tend to restrain disclosure.
324 Analyzing the impact of shareholder composition on corporate voluntary disclosure is important for understanding
325 how shareholder type and composition affect disclosure strategies. Based on these studies, hypothesis H5 expects a
326 significant relationship to exist between shareholder composition and the extent of voluntary disclosure.

327

##### 328 H5: Shareholder composition determines the extent of voluntary disclosure.

329

330 Based on these previous studies, this study examines the impact of corporate financial indicators, corporate
331 characteristics, listing market, industry classification, and shareholder composition on the voluntary disclosure
332 behavior of Japanese Companies.

333

# 334 3. Research methods

## 335 3.1 Subject of research

336 The target of this study is all Companies listed on the Tokyo Stock Exchange and regional securities markets in
337 Japan. This enables a comprehensive analysis of all listed companies in Japan as a whole.

338

## 339 3.2 Samples used

340 The analysis period of this study is the quarterly data from March 2009 to March 2024, and the total number of
341 listed companies during this period is 5,915. The reason for using 2009 as the starting point is that the establishment
342 of the International Integrated Reporting Council (IIRC) was proposed by the Prince of Wales in December of the
343 same year [47]. The reason for this change is that the establishment of the IIRC (International Integrated Reporting
344 Council) was proposed by the Prince of Wales in a speech in December of that year. This report analyzes what changes
345 have occurred in the disclosure of information by Japanese companies since the proposal of this integrated reporting
346 concept. The data (the table before regression) for reproducing the results of this experiment is provided as
347 Supporting information (S1). 348

## 349 3.3 Research model

350 The research model is outlined below, and each variable and other information is explained below shown in Fig5.

351

352	Fig 5. Research Framework

353

## 354 3.4 Variable

### 355 3.4.1 Object variable

356 The Object variable in this study is Voluntary Reporting (VR), which indicates whether a Company prepares a
357 voluntary disclosure report. It is defined as a dummy variable that is 0 if the Company does not disclose a voluntary
358 disclosure report and 1 if it does. We collected PDF data by examining each Company's website, financial reports,
359 and other publicly available information to see if the Company had a voluntary disclosure report. Although the name
360 of the report may change from year to year, this study included in the analysis whether or not a report containing
361 financial and non-financial information was issued, regardless of the name shown in Fig 1.

362

### 363 3.4.2 Explanation variable

| 364 | Explanation variables are set (60 in total) in five categories of factors that affect voluntary disclosure. Detailed |
| --- | --- |
| 365 | definitions of these variables and data processing methods (e.g., log transformations, dummy variable conversion, |
| 366 | etc.) are provided **in S2.** |
| 367 | - H1: 9 financial indicators (β1_Financial Indicators) (β1-1 to β1-9) |
| 368 | - H2: 8 β2_Company Characteristics (β2-1 to β2-8) |
| 369 | - H3: Listed Market (β3_Market-Related Factors) (β3-1 to β3-4) 4 |
| 370 | - H4: β4_Industry Classification (β4-1 to β4-33) 33 |
| 371 | - H5: Shareholder Composition (β5_Shareholding Ratios) (β5-1 to β5-5) 5 |
| 372 | - Point in time information (quarterly variable: β6_ Quarter) |
| 373 |  |
| 374 | **3.5 Analysis Model** |
| 375 | **3.5.1 Model expression** |
| 376 | Binomial logistic regression analysis is employed to predict whether or not a company will prepare a voluntary |
| 377 | disclosure report. This technique is appropriate when the objective variable takes on a binary value (0 or 1) and allows |

378 for a probabilistic estimation of the impact of the Explanation variable on the Objective variable. By using a logistic
379 function, probabilities between 0 and 1 can be predicted from the weighted sum of the Explanation variables. In this
380 study, we analyze how financial indicators, Company characteristics, listing market, industry classification,
381 shareholder composition, and point in time information (quarterly) affect the voluntary disclosure behavior of
382 Companies. The present model is expressed as follows:

𝑖=1

𝐽=1

𝑘=1

𝑙=1

383

𝑙𝑜𝑔𝑖𝑡(𝑃(𝑉𝑅 = 1)) = 𝛽_0_ + ∑^9^

β1 − 𝑖 ∙ 𝑥_1−𝑖_ +∑^8^

β2 − 𝑗 ∙ 𝑥_2−𝑗_ +∑^4^

β3 − 𝑘 ∙ 𝑥_3−𝑘_ +∑^33^

β4 −

384

𝑙 ∙ 𝑥_4−𝑙_+∑^5^

β5 − 𝑚 ∙ 𝑥_5−𝑚_+Β6⋅ Quarter+ϵ

385 Here, VR is the Object variable and is set to 1 if the company issues voluntary disclosure reports and 0 if it does not.

𝑚=1

386 In addition, the Explanation variable XXX falls into the five categories described above.

387

### 388 3.5.2 Analytical method

#### 389 3.5.2.1 Binomial logistic regression

390 In this study, a binomial logistic regression was used to predict whether a Company would issue a voluntary
391 disclosure report. This method is appropriate when the Object variable is binary (0 or 1) and shows the effect of a
392 change in the Explanation variable on the probability of occurrence. The estimated coefficients (β values) indicate
393 the impact of the Explanation variable on the probability of any disclosure. Specifically, it is interpreted as follows:

394

395 ⚫	The regression coefficient (Coef) represents the logarithm of the odds ratio for a one-unit increase in the
396 Explanation variable.
397 ⚫	Odds (Odds) is the ratio of the probability that a company will make a voluntary disclosure to the probability
398 that it will not. If the odds ratio is greater than 1, the probability of voluntary disclosure increases.

399

𝑂𝑑𝑑𝑠 = 𝑃(𝑉𝑅=1)

𝑃(𝑉𝑅=0)

The term is represented by the following

400

401

402

- The probability is the probability that a company will make a voluntary disclosure. It is derived from the odds ratio by the following equation:.

P(VR=1) =	^1^

1+𝑒 −𝑙𝑜𝑔𝑖𝑡(𝑃(𝑉𝑅=1)

| 403 |  |
| --- | --- |
| 404 | **3.5.2.2 Visualization by sigmoid function** |
| 405 | To make the results of the logistic regression more intuitive, the influence of the Explanation variable was |
| 406 | visualized using a sigmoid function. This function shows the variation in arbitrary disclosure probability due to |
| 407 | changes in the Explanation variable as an S-shaped curve, which is classified into three main patterns |
| 408 | - **Rising right:** the probability of voluntary disclosure increases as the Explanation variable increases. |
| 409 | - **Rightward**: the probability of voluntary disclosure declines as the Explanation variable increases. |
| 410 | - **Other**: Explanation variable shows no clear effect on voluntary disclosure. |
| 411 | The sigmoid function is shaped by the positive and negative regression coefficients (Coef) and shows a sharp change |
| 412 | in the middle part of the curve as the odds (Odds) change. This visualization provides a visual understanding of how |
| 413 | the Explanation variable is affecting the voluntary disclosure. |

414

#### 415 3.5.2.3 Coefficients, odds, and probability correlations

416 In logistic regression, regression coefficients, odds, and probabilities are closely related. Therefore, how each
417 Explanation variable affects the probability of voluntary disclosure is also analyzed in terms of regression coefficients
418 (Coef) and odds (Odds). When the regression coefficients are positive, the odds exceed 1, the sigmoid curve rises
419 steadily, and the probability of voluntary disclosure increases. When the regression coefficient is negative, the odds
420 are less than 1, the sigmoid curve falls steadily, and the probability of voluntary disclosure decreases.

421

#### 422 3.5.2.4 Validation of analytical methods

423 In this study, to avoid multicollinearity, the independence of each variable was verified using VIF (variance
424 expansion factor), and the model was built to the extent that there were no problems. In addition, training and test
425 data were fairly distributed by data partitioning to prevent overtraining of the model and ensure generality of the
426 results.

427

#### 428 3.5.2.5 Response to multicollinearity

429 Since a large number of Explanation variables are used in this model, there is concern about the occurrence of
430 multicollinearity. To address this, the VIF (variance expansion factor) was calculated and multicollinearity was
431 examined for Explanation variables with a VIF greater than ten. In particular, for β3_Market, β4_Sector, and
432 β5_Shareholding Ratio, the Explanation variables with the highest frequency (e.g., β3-1(P): Prime Market, β4-6-25:
433 Information and Communication Industry, β5-4: Shareholder Composition_Personal and Others) were used as
434 reference categories and deducted before analysis. The VIF is deducted prior to the analysis. Other Explanation
435 variables with VIF greater than 10 (e.g., total stock market capitalization, total assets, etc.) were retained as
436 explanation variables after considering their content and determining that they were not multicollinear (see S3, Table
437 3-1 for details).

438

### 439 3.5.3 Response to data splitting

440 Data partitioning is important to avoid over-learning and to improve generalization ability in model construction
441 and evaluation. In this study, data are split by Company ID to take into account the different characteristics of each
442 Company. This prevents information from crossing across Companies and improves the generalization performance
443 of the model.
444 Specifically, the following procedure was used to split the data
445 1.	Split by company ID: Group data by company ID and split them into training and test data. This method prevents
446 data crossover between different companies and enables more accurate model evaluation.
447 2.	Split training and test data: 80% of the total data is used as training data and the remaining 20% as test data. In
448 this way, the model is trained on the training data and the performance of the model is evaluated on the test data.
449 3.	Confirmation of the balance of the split: Check that the number of Companies and data are distributed in a
450 balanced manner, including industry sectors. This ensures unbiased data partitioning and fair model evaluation.

451

# 452 4. Empirical results

## 453 4.1 Overall model evaluation

454 The results of the logistic regression analysis confirm that the model is fully accurate. The overall correctness rate
455 (Accuracy) is 89.18%, which accurately predicts the presence of voluntary disclosure (see S3, Table 3-1). The AUC
456 (area under the ROC curve) was as high as 0.9449, indicating excellent ability to distinguish between voluntary
457 (VR=1) and involuntary (VR=0) disclosures (see S3, Table 3-4).
458 The Confusion Matrix results show that 33,826 "involuntary disclosures (VR=0)" were correctly predicted with
459 96% accuracy, while 5,840 "voluntary disclosures (VR=1)" were correctly predicted with 64% accuracy, and 3,326
460 were incorrectly labeled "involuntary disclosures" (see S3, Table 3-2). Furthermore, according to the Classification
461 Report, the compliance rate for "Non-Voluntary Disclosure (VR=0)" was 0.91, the reproducibility rate was 0.96, and
462 the F1 score was extremely high at 0.93. In contrast, for "Voluntary Disclosure (VR=1)," the fit rate is 0.80, the
463 repeatability is 0.64, and the F1 score is 0.71, which are slightly lower, but still retain adequate performance (see S3,
464 Table 3-3).

465

## 466 4.2 Influence analysis of Explanation variable

467 The coefficients, standard errors, and P-values of the Explanation variables were analyzed to test the significance
468 and direction of the impact of each on voluntary disclosure (see S4). As a result, there was a clear distinction between
469 the hypotheses between Explanation variables that have a positive impact on voluntary disclosure and those that have
470 a negative impact shown in Fig6.

471

472 Fig 6. Logistic Regression Coefficients Grouped by Hypothesis
473 This figure shows the logistic regression results grouped by the corresponding hypotheses (H1-H5) and includes a time element (Quarter, 6). Each section
474 is separated by a dashed line to indicate the boundary between hypotheses. H1: Financial Indicators, H2: Company Characteristics, H3: Market-Related
475 Factors, H4: Industry Classification, H5: Shareholding Ratios, and Time Element (Quarter). The target variable is whether a company discloses voluntarily
476 or not.

477

## 478 4.3 Results of visualization by sigmoid function

479	To visually understand how Explanation variables affect voluntary disclosure behavior, we visualized the results

480	with a sigmoid function shown in Fig7.

481

482	Fig 7. Combined Upward, Downward, and Other Sigmoid Curves

483	This figure combines three types of sigmoid curves: upward, indicating a positive relationship; downward, indicating a negative relationship; and other,

484	indicating minimal or no relationship with voluntary disclosure.

485

486 •	A steadily rising sigmoid function indicates that the probability of voluntary disclosure increases as the
487 Explanation variable increases, indicating that Companies tend to disclose information proactively. For example,
488 market value and number of employees fall into this category.
489 •	A steadily declining sigmoid function indicates that the probability of voluntary disclosure decreases as the
490 Explanation variable increases, indicating a tendency for Companies to restrain disclosure; this is the case for PER
491 and foreign market listings.
492 •	Other sigmoid functions represent cases where Explanation variables do not show a clear impact on
493 voluntary disclosures. Operating income and dividends fall into this category.
494 These results show that different Explanation variables have different effects on voluntary disclosure behavior and
495 clearly indicate how each variable acts on voluntary disclosure based on the form of the sigmoid function. The
496 following sections summarize the results of the analysis for each hypothesis and clarify points to be considered in
497 the discussion.

498

### 499 4.3.1 Hypothesis H1: The impact of Financial Indicators on voluntary

500	**disclosure.**

501

502	Market value (β1-1), total assets (β1-2), and capitalization (β1-3) increased the likelihood of voluntary disclosure,

503	as expected shown in Fig8-1.

504

505 Fig8-1. H1: Results of Sigmoid Function for the Impact of Financial Indicators on Voluntary Disclosure
506 This figure illustrates the impact of financial indicators on voluntary disclosure. The sigmoid function demonstrates a positive relationship between
507 Market Value, TTLASSET, CAPITAL, and Sales, indicating that an increase in these variables is associated with a higher probability of voluntary
508 disclosure. In contrast, PER shows a negative relationship, and variables such as ISSUEDSHARE, Operating Profit, PBR, and DPS have no significant
509 effect.

510

511 These results are consistent with previous studies	[15,16] that larger Companies are more active in disclosure.
512 We also confirm that sales (β1-5), contrary to expectations, promote voluntary disclosure. Operating profit (β1-6)
513 and PBR (β1-7) were statistically significant but had limited impact on voluntary disclosure, while PER (β1-8)
514 confirmed a "right-sided" relationship that suppressed voluntary disclosure, indicating that Companies with higher
515 price-to-earnings ratios tend to suppress information disclosure. As a new finding, this result indicates that Companies
516 with higher PERs may suppress disclosure; no statistically significant effects were found for ISSUEDSHARE (β1-
517 4) or DPS (β1-9). Detailed results are presented in S5.

518

### 519 4.3.2 Hypothesis H2: The impact of Company Characteristics on voluntary

520	**disclosure.**

521

522	Employee headcount (β2-1), GAAP (β2-4), ISO 45001 (β2-7), and ISO 14001 (β2-8) promoted voluntary

523	disclosure, consistent with previous studies ([23,22,29]) shown in Fig8-2.

524

525	Fig8-2. H2: Results of Sigmoid Function for the Influence of Corporate Characteristics on Voluntary Disclosure

526	This figure shows the influence of company characteristics on voluntary disclosure. A positive relationship is seen with Employee Count, GAAP,

527	ISO45001, and ISO14001, suggesting that these factors increase the likelihood of disclosure. Foreign Market and Stock Repurchase exhibit negative

528	relationships, while Years since Establishment and Listing Period show no significant impact.

529

530	On the other hand, Foreign Market (β2-5) and Stock Repurchase (β2-6) showed results that suppressed voluntary

531	disclosure and differed from previous studies that found foreign market listings promote disclosure. Years since

532	Establishment (β2-2), Listing Period (β2-3) are statistically significant but have no significant effect on voluntary

533	disclosure. Detailed results are presented in S5.

534

### 535 4.3.3 Hypothesis H3: The impact of Market-Related Factors on voluntary

536 **disclosure**
537 **Hypothesis H3** examined the impact of listing market on voluntary disclosure by Companies. We confirmed that
538 Companies listed on the Standard (β3-2) and Growth (β3-3) markets are less likely to engage in voluntary disclosure
539 than Companies listed on the Prime market shown in Fig8-3.

540

541	Fig8-3. H3: Results of Sigmoid Function for the Impact of Listing Market Factors on Voluntary Disclosure

542	This figure displays the impact of listing market on voluntary disclosure. The sigmoid function reveals that companies listed in the Standard, Growth,

543	and Local markets are less likely to voluntarily disclose information compared to those in the Prime market.

544

545	These results confirm that the more regulated the market, the more transparent the disclosure with previous study

546	[39]. For local markets (β3-4), no statistically significant effects were identified, suggesting that there is no significant

547	impact on voluntary disclosure. Detailed results are provided in S5.

548

### 549 4.3.4 Hypothesis H4: The impact of Industry Classification on voluntary

550 **disclosure**
551 **Hypothesis H4** examines the impact on voluntary disclosure by Companies in 33 industries, which is a more
552 detailed breakdown of the industry categories (10 major industries). In the analysis, Information & Communication
553 (β4-6-25) was used as the reference category, and the results of the analysis for the 33 industries showed that the
554 impact on voluntary disclosure varies significantly by industry shown in Fig8-4.

555

556	Fig8-4. H4: Results of Sigmoid Function for the Effect of Industry Classification on Voluntary Disclosure

557	This figure represents the influence of industry classification on voluntary disclosure. Positive relationships are observed in industries such as Insurance,

558	Nonferrous Metals, Pharmaceuticals, Air Transportation, Chemicals and Banking, while negative relationships are evident in sectors such as Oil,

559	Agriculture, Retail. Some industries, including Construction and Machinery, show no significant effect on disclosure.

560

561	Due to the wide variety of industries, we adjusted S5 to examine each industry and prepared the following Table

562	2.

563 Table 2: Results of Hypothesis H4 - Impact of Industry Classification on Voluntary Disclosure

| **Reference** | **10 major industry categories** | **Assumed** | **33 industry subcategories (Explanatory Variable)** | **Observed** | **statistically**  **Significant* (NS: Not statistically significant (NS: Not statistically significant)** | **Result** | **Unexpected Findings** |
| --- | --- | --- | --- | --- | --- | --- | --- |
| Β 4-1-1 | Fishery, Agriculture & Forestry | →↘ | Fishery, Agriculture & Forestry | ↘ | * | ↘ | - |
| Β 4-2-2 | Mining | →↘ | Mining | ↘ | NS | - | - |
| Β 4-3-3 | Construction | →↘ | Construction | ↗ | NS | - | - |
| Β 4-4-4 | Manufacturing | ↗ | Foods | ↘ | * | ↘ | Yes |
| Β 4-4-5 |  | ↗ | Textiles and Apparels | ↘ | * | ↘ | Yes |
| Β 4-4-6 |  | ↗ | Pulp and Paper | → | NS | - | - |
| Β 4-4-7 |  | ↗ | Chemicals | ↗ | * | ↗ | - |
| Β 4-4-8 |  | ↗ | Pharmaceutical | ↗ | * | ↗ | - |
| Β 4-4-9 |  | ↗ | Oil and Coal Products | ↘ | * | ↘ | Yes |
| Β 4-4-10 |  | ↗ | Rubber Products | ↗ | * | ↗ | - |
| Β 4-4-11 |  | ↗ | Glass and Ceramics Products | ↘ | * | ↘ | Yes |
| Β 4-4-12 |  | ↗ | Iron and Steel | ↘ | * | ↘ | Yes |
| Β 4-4-13 |  | ↗ | Nonferrous Metals | ↗ | * | ↗ | - |
| Β 4-4-14 |  | ↗ | Metal Products | ↘ | * | ↘ | Yes |
| Β 4-4-15 |  | ↗ | Machinery | → | NS | - | - |
| Β 4-4-16 |  | ↗ | Electric Appliances | → | NS | - | - |
| Β 4-4-17 |  | ↗ | Transportation Equipment | ↘ | * | ↘ | Yes |
| Β 4-4-18 |  | ↗ | Precision Instruments | ↗ | * | ↗ | - |
| Β 4-4-19 |  | ↗ | Other Products | ↘ | * | ↘ | Yes |
| Β 4-5-20 | Electricity, Gas | →↘ | Electric Power and Gas | ↘ | * | ↘ | - |
| Β 4-6-21 |  | ↗ | Land Transportation | ↘ | * | ↘ | Yes |
| Β 4-6-22 | Transport, Information and communications | ↗ | Marine Transportation | ↗ | * | ↗ | - |
| Β 4-6-23 |  | ↗ | Air Transportation | ↗ | * | ↗ | - |
| Β 4-6-24 |  | ↗ | Warehousing and Harbor Transportation | ↘ | * | ↘ | Yes |
| Β 4-6-25 |  | ↗ | Information & Communication | ↗ | Baseline | - | - |
| Β 4-7-26 | Commercial | ↗ | Wholesale Trade | ↘ | * | ↘ | Yes |
| Β 4-7-27 |  | ↗ | Retail Trade | ↘ | * | ↘ | Yes |
| Β 4-8-28 | Finance and Insurance | ↗ | Banks | ↗ | * | ↗ | - |
| Β 4-8-29 |  | ↗ | Securities and Commodities Futures | ↘ | * | ↘ | Yes |
| Β 4-8-30 |  | ↗ | insurance | ↗ | * | ↗ | - |
| Β 4-8-31 |  | ↗ | Other Financing Business | ↗ | * | ↗ | - |
| β 4-9-32 | Real Estate | →↘ | Real Estate | ↘ | * | ↘ | - |
| β 4-10-33 | Services | ↗ | Services | ↘ | * | ↘ | Yes |

564 Below are the results for major industries.

##### 565 1. Fishery, Agriculture & Forestry: New findings: None

566 One medium classification included in Fishery, Agriculture & Forestry confirmed the negative impact of voluntary
567 disclosure as predicted.

##### 568 2. Mining: new findings: none

569 No statistically significant results were obtained for the one medium classification included in Mining.

##### 570 3.Construction: New findings: None

571 No statistically significant results were obtained for the one medium classification included in Construction.

##### 572 4. Manufacturing: new discoveries: including

573 The largest number of medium-sized industries included in Manufacturing was 16, of which there were three that
574 were not statistically favorable. Although our initial expectation was that the respondents would be more willing to
575 voluntarily disclose, only five industries (β4-4-7: Chemicals, β4-4-8: Pharmaceuticals, β4-4-10: Rubber Products,
576 β4-4-13: Nonferrous Metals, and β4-4-18: Precision Instruments) were more willing to do so, while eight industries
577 were less willing. and β4-4-18: Precision Instruments), and only eight industries (β4-4-4: Foods, β4-4-5: Textiles and
578 Apparels, β4-4-9: Oil and Coal Products, β4-4-11: Glass and Ceramics Products, β4-4-12: Glass and Ceramic
579 Products, and β4-4-13: Precision Instruments). Ceramics Products, β4-4-12: Iron and Steel, β4-4-14: Metal Products,
580 β4-4-17: Transportation Equipment, and β4-4-19: Other Products), and the results were negative. The results of the
581 survey revealed new findings that were not apparent from the increase in the

##### 582 5. Electricity, Gas: New findings: None

583 One medium classification included in Electricity, Gas Fishery, Agriculture & Forestry confirmed the negative
584 impact of voluntary disclosure as predicted.

##### 585 6. Transport, Information and Communications: new discoveries: including

586 There are five subcategories in the Transport, Information and Communications category, and for the four
587 subcategories other than the reference category, two industries (Β 4-6-22: Marine Transportation and Β 4-6-23: Air
588 Transportation) were proactive in voluntary disclosure, as expected, while the remaining two industries (Β 4-6-21:
589 Land Transportation and Β 4-6-24: Warehousing and Harbor Transportation) were reactive. Air Transportation), while
590 the remaining two (Β 4-6-21: Land Transportation and Β 4-6-24: Warehousing and Harbor Transportation) were not.
591 The results of the two industries (β4-6-21: Land Transportation and β4-6-24: Warehousing and Harbor
592 Transportation) showed a negative trend.

##### 593 7. Commercial: new findings: including

594 Although we had expected positive effects on voluntary disclosure in two of the medium categories included in
595 the Commercial category (β4-7-26: Wholesale Trade and β4-7-27: Retail Trade), we found negative effects on
596 voluntary disclosure in both categories. This is a different result from other industries, where there is a mixture of
597 positive and negative voluntary disclosure, and a new finding was detected that could not be determined by the
598 increasing trend in the major categories.

##### 599 8. Finance and Insurance: new findings: including

600 Finance and Insurance included four subcategories, three of which (β4-8-28: Banks, β4-8-30: Insurance, and β4-
601 8-31: Other Financing Business) were active in voluntary disclosure, as expected, while the remaining one (β4-8-29:
602 Securities and Commodities Futures) was passive. The remaining one (β4-8-29: Securities and Commodities Futures)
603 was negative, revealing new insights that could not be gleaned from the general classification of increasing trends.

##### 604 9. Real Estate: New Findings: None

605 One medium classification included in Real Estate confirmed the negative impact of voluntary disclosure as
606 predicted.

##### 607 10. Services: new findings: including

608 Contrary to the initial prediction that one medium classification (β4-10-33: Services) included in Services would
609 have a positive impact on voluntary disclosure, a negative impact was identified, which is considered a new finding.

610

611 Overall, the impact of industry classification on the voluntary disclosure behavior of Companies varies from
612 industry to industry and may require consideration of the severity of regulations and horizontal practices in the
613 industry. Explanation variables that were found to diverge from previous studies will be subject to additional
614 consideration in the Discussion section, based on their peculiarities in Japan. Detailed results by industry will be
615 provided in S5.

616

### 617 4.3.5 Hypothesis H5: The impact of Shareholding Ratios on voluntary

618	**disclosure.**

619

620	The impact of shareholder composition on voluntary disclosure is diverse. The shareholding by financial

621	institutions (β5-1) promotes voluntary disclosure, consistent with Rouf & Harun (2010) shown in Fig8-5.

622

623	Fig8-5. H5: Results of Sigmoid Function for the Influence of Ownership Structure on Voluntary Disclosure

624	This figure illustrates the impact of shareholding structure on voluntary disclosure. The analysis shows that higher ownership by financial institutions

625	increases the likelihood of disclosure, whereas ownership by other corporations is associated with a lower probability of disclosure. No significant effect

626	is found for miscellaneous entities and foreign corporations.

627

628	On the other hand, other corporations (β5-2) showed a tendency to discourage disclosure, a result that differs from

629	previous studies. Foreign corporations (β5-3) and individuals other (β5-5) had no effect on voluntary disclosure.

630	Detailed results are presented in S5.

631

632	The results for the time point information (β6), which was added as an Explanation variable this time, are shown in

633	Fig8-6 below.

634

635	Fig8-6. Results of Sigmoid Function for the Influence of Other Factors on Voluntary Disclosure

636	This figure shows the influence of the quarter on voluntary disclosure. The analysis suggests that temporal factors may play a role in disclosure behavior,

637	but the results vary depending on specific conditions.

638

639	Although the quarter's point in time was replaced by a dummy variable for convenience, this study suggests that it

640	has no effect on voluntary disclosure.

641

642 Based on the results of our previous analysis, we will proceed with a discussion of the impact of company size,
643 financial indicators, company characteristics, listing market, industry classification, and shareholder composition on
644 voluntary disclosure behavior. Detailed discussion will be based on the following points
645 1.	**Impact of Financial Indicators**: While a company's size and profitability act as drivers of voluntary
646 disclosure, some indicators, such as PER, have a negative impact on disclosure. The implications of these
647 results and why each indicator has a different impact will be discussed.
648 2.	**Impact of Company Characteristics**: While the number of employees, adoption of GAAP, and acquisition
649 of ISO promote voluntary disclosure, foreign market listings and share repurchases discourage voluntary
650 disclosure. We will examine what factors are behind this trend.
651 3.	**Impact of Listed Markets**: as originally planned, but discussion of the impact of different market
652 regulations and expectations on voluntary disclosure will focus on the differences between prime and other
653 markets.
654 4.	**Impact of Industry Classification**: Certain industries are more active in voluntary disclosure, while others
655 are more reluctant. This section examines how the characteristics of each industry affect disclosure behavior.
656 5.	**Impact of shareholder composition**: Discuss why the shareholder composition of financial institutions
657 promotes voluntary disclosure while other corporations discourage voluntary disclosure.

658

659	**5. Discussion**

## 660 5.1 On the development of model equations

661	In this study, we analyzed the factors affecting voluntary corporate disclosure by dividing them into five categories:

662	financial indicators, corporate characteristics, listed market, industry classification, and shareholder composition. The

663	initial model equation was expressed as follows:

𝑖=1

𝐽=1

𝑘=1

𝑙=1

664

𝑙𝑜𝑔𝑖𝑡(𝑃(𝑉𝑅 = 1)) = 𝛽_0_ + ∑^9^

β1 − 𝑖 ∙ 𝑥_1−𝑖_ +∑^8^

β2 − 𝑗 ∙ 𝑥_2−𝑗_ +∑^4^

β3 − 𝑘 ∙ 𝑥_3−𝑘_ +∑^33^

β4 −

665

𝑙 ∙ 𝑥_4−𝑙_+∑^5^

β5 − 𝑚 ∙ 𝑥_5−𝑚_+β6⋅ Quarter+ϵ

666 where each β corresponds to each Explanation variable and X represents the observed value of each variable.

𝑚=1

667 However, based on the post-experimental results, it became clear that the Explanation variable could be categorized
668 into three main sigmoidal forms: **rising right**, **falling right**, and other (flat or atypical shape). This indicated that the
669 model equations can be aggregated as follows:
670 logit(P(VR=1))=β0+βupward ⋅ Upward Factors+βdownward ⋅ Downward Factors+βother ⋅ Other Factors+βquarter ⋅
671 Quarter+ϵ
672 WHEREAS:
673 •	**Upward Factors** are factors that promote voluntary disclosure, such as Market Value, Total Assets, Capital
674 Stock, Sales, Employee Count, GAAP, ISO 45001, and ISO 14001.
675 •	**Downward Factors** are factors that inhibit voluntary disclosure, such as Foreign Market, PER, Stock
676 Repurchase, MARKET(S), and MARKET(G).
677 •	**Other Factors** are factors such as ISSUEDSHARE, Operating Profit, PBR, and DPS that are not
678 statistically significant or have no clear impact.
679 This aggregation has resulted in a simple and effective restructuring of the original complex model, making it
680 easier to visually understand the effects of each Explanation variable. Next, we proceed with a discussion of how
681 these factors affect the voluntary disclosure behavior of companies, based on each hypothesis.

682

## 683 5.2 Discussion of each hypothesis

684 Table 3 summarizes the results of the analysis of each Explanation variable.

##### 685 Table3_ Summary of Experimental Results

|  | H1 | H2 | H3 | H4 | H5 | - | TTL |
| --- | --- | --- | --- | --- | --- | --- | --- |
|  | Financial Indicators | Company Characteristics | Market-.  Related Factors | Industry Classification | Shareholding Ratios | Time  point Information |  |
| Reference | β 1 | β 2 | β 3 | β 4 | β 5 | β 6 |  |
| Explanatory Variables | 9 | 8 | 4 | 33 | 5 | 1 | 60 |
| Base line | - | - | △1 | △1 | △1 | - | △3 |
| Not statistically significant | weak2 | - | △1 | △5 | weak2 | - | △10 |
| **Sub-TTL** | **7** | **8** | **2** | **27** | **2** | **1** | **47** |
| Result: Upward | 4 | 4 | - | 10 | 1 | - | 19 |
| Result: Downward | 1 | 2 | 2 | 17 | 1 | - | 23 |
| Result: Other | 2 | 2 | - | - | - | 1 | 5 |
| Unexpected Findings | 4 | 4 | - | 15 | 1 | - | 24 |

686 In the following, a discussion will be conducted on Explanation variables for which results that differ from
687 predictions based on prior studies and other sources are detected.

688

### 689 5.2.1Hypothesis H1: The impact of financial indicators on voluntary disclosure

690 With regard to financial indicators, β1-5 (Sales), β1-6 (Operating Profit), β1-7 (PBR), and β1-8 (PER) yielded
691 results that differ from the forecasts. A discussion of each variable is presented below.
692 1. **β**1-5**: Sales**
693 Sales (β1-5), whose negative impact on voluntary disclosure had been verified in previous studies, was confirmed
694 to have a positive impact in the present experimental results, on the contrary. As was observed in the trends of other
695 Explanation variables, this confirms the trend that larger Companies are more proactive in disclosure, which is not
696 far above the expected range.

##### 697 2. β1-6: Operating Profit

698 With respect to Operating Profit, some prior studies showed an association with positive voluntary disclosures,
699 while others showed negative results. Operating Profit was statistically significant, but the impact was limited. It is
700 suggested that short-term earnings fluctuations may make it difficult for Companies to implement a consistent
701 disclosure strategy.

##### 702 3. β1-7: Price-to-book ratio (PBR)

703 PBR is an indicator to evaluate the market value of a company, since it is calculated by dividing the stock price
704 per share by the net assets per share. In this study, too, PBR showed statistically significant results, but the impact
705 was negligible. It is possible that Companies with higher market values are more trusted and do not feel the need for
706 additional disclosure.

##### 707 4. β1-8: Price-to-Earnings Ratio (PER)

708 Since PER is calculated by dividing the stock price per share by earnings per share, a high PER value is an indicator
709 that reflects a company with high growth expectations. Normally, a company with a high PER would be highly valued
710 by the market, and as a result, disclosure would be more important.

711

### 712 5.2.2Hypothesis H2: The impact of corporate characteristics on voluntary

713 **disclosure**
714 With respect to Company characteristics, the results differed from the predictions in β2-2 (Years since
715 Establishment), β2-5 (Foreign Market), β2-7 (ISO 45001), and β2-8 (ISO 14001). A discussion of each variable is
716 presented below.
717 **1.** β**2-2:** Years **since Establishment**
718 The effect of Company age was limited and consistent with previous studies ( [25,26,24]).

##### 719 2.β2-5: Foreign Market

720 In prior studies, it was predicted that Companies relying on foreign markets would be more willing to make
721 voluntary disclosures in order to respond to different regulatory environments and investor expectations. However,
722 in this study, Foreign Market resulted in less voluntary disclosure, a new finding that contradicts expectations. It is
723 possible that the Foreign Market is taking a cautious disclosure strategy into consideration of disclosure standards
724 and risks.

| 725 | **3. β2-7: ISO45001** |
| --- | --- |
| 726 | ISO 45001 is a certification for occupational health and safety, and this study found that the acquisition of ISO |
| 727 | 45001 has a positive impact on voluntary disclosure. Unlike previous studies, this result is a new finding: through |
| 728 | the acquisition of ISO 45001, social responsibility may be emphasized. |
| 729 | **4. β2-8: ISO14001** |
| 730 | ISO 14001 is a certification for environmental management, and this study confirms that the acquisition of ISO |
| 731 | 14001 has a positive impact on voluntary disclosure. This is a new finding, as it differs from the results of previous |
| 732 | studies, and it is thought that the intention is to actively promote environmental responsiveness and enhance social |
| 733 | reputation through ISO 14001. |
| 734  735 | **5.2.3Hypothesis H3: The impact of the listing market on voluntary disclosure** |
| 736 | Hypothesis H3 examines the impact of listing market on voluntary disclosure. The experimental results were in |
| 737 | line with the hypothesis, confirming that Companies listed on the **Standard (β3-2**), **Growth (β3-3**), and Regional |
| 738 | (β3-4) markets are more reluctant to make voluntary disclosures than Companies listed on the Prime market . This |
| 739 | result is consistent with previous studies and no new findings were found in particular. |
| 740 |  |
| 741 | **5.2.4Hypothesis H4: The impact of industry classification on voluntary disclosure** |
| 742 | Industry-specific differences were identified, with results differing from forecasts in certain industries. The |
| 743 | following is a discussion of these findings. |
| 744 | 1. Manufacturing |
| 745 | In the manufacturing sector, Chemicals (β4-4-7), Pharmaceuticals (β4-4-8), Rubber Products (β4-4-10), |
| 746 | Nonferrous Metals (β4-4-13), and Precision Instruments (β4-4-18) positively impacted voluntary disclosures as |
| 747 | expected. Foods (β4-4-4), Textiles and Apparels (β4-4-5), Oil and Coal Products (β4-4-9), Glass and Ceramics |
| 748 | Products (β4-4-11), Iron and Steel (β4-4-12), Metal Products (β4-4-14), Transportation Equipment (β4-4-17), and |
| 749 | Other Products (β4-4-19) were reluctant. Particularly in the Food and Oil & Coal industries, it is possible that |
| 750 | environmental issues and regulatory compliance may have inhibited disclosure. |
| 751 | 2. Transport, Information and Communications |
| 752 | Marine Transportation (β4-6-22) and Air Transportation (β4-6-23) were active, while Land Transportation (β4-6-21) |
| 753 | and Warehousing and Harbor Transportation (β4 -6-24) were passive. In Land Transportation, labor issues and safety |
| 754 | may have inhibited disclosure, while in Warehousing and Harbor Transportation, confidentiality of trading terms may |
| 755 | have influenced disclosure. |
| 756 | 3. Commercial |
| 757 | Wholesale Trade (Β 4-7-26) and Retail Trade (Β 4-7-27) were, contrary to expectations, reluctant. Retail Trade |
| 758 | in particular may have been reluctant to disclose in order to avoid the risk of information leakage in a highly |
| 759 | competitive market. |
| 760 | 4. Finance and Insurance |
| 761 | Securities and Commodities Futures (Β 4-8-29) was reluctant, while Banks (Β 4-8-28), Insurance (Β 4-8-30), and |
| 762 | Other Financing Business (Β 4-8-31) were active. Uncertainty and regulation of financial instruments transactions |

763	are thought to be restraining factors for disclosure.

764	5.	Services

765	Services (β4-10-33) was, contrary to expectations, reluctant. Services may be cautious about disclosure due to the

766	intangible nature of the industry and the particular nature of the competitive environment.

767

### 768 5.2.5Hypothesis H5: The impact of shareholder composition on voluntary

769 **disclosure**
770 The results of the study of the impact of shareholder composition confirmed the tendency for voluntary disclosure
771 to be more reluctant, especially for companies with a high percentage of "Other Corporation" ownership. The
772 following is a discussion of the results.

##### 773 1. β5-2: Ownership Percentage of Other Corporations

774 It is suggested that Companies with a high percentage of ownership by corporate shareholders may be more
775 cautious about information disclosure and may intend to minimize information sharing for strategic reasons. It is
776 possible that among corporate shareholders, information is shared only internally or among shareholders, and there
777 is less need for broad market disclosure. Furthermore, corporate shareholders are likely to be reluctant to disclose
778 information to avoid the risk of undermining their competitive advantage, and they may have less need to seek
779 additional public information because of their ability to communicate directly with management.

780

781 One limitation of the study is that the use of a large number of Explanation variables limited the detailed analysis
782 for each hypothesis. We also recognize as a limitation that we were unable to fully take into account the differences
783 in the content and format of the reports, which are determined arbitrarily by the Companies. Furthermore, the fact
784 that the time variable was treated as a dummy variable is also an issue, as the characteristics of the panel data could
785 not be fully evaluated.

786

787	**6. Conclusion**

788 In this study, we analyzed the voluntary disclosure behavior of Japanese listed companies from various
789 perspectives and made several important findings. In particular, based on the hypothesis that financial indicators
790 influence the degree of voluntary disclosure (referred to as Hypothesis 1 (H1) in this paper), we clearly showed
791 differences in the effects of price-to-book ratio (P/B ratio) and price-to-earnings ratio (P/E ratio) on disclosure
792 behavior. Based on the hypothesis that corporate characteristics influence the degree of voluntary disclosure
793 (Hypothesis 2 (H2)), we identified differences in the impact of whether the company is listed on a foreign market or
794 has obtained ISO certification. Furthermore, based on the hypothesis that the listing market influences the degree of
795 voluntary disclosure (Hypothesis 3 (H3)), the effect of differences in listing market on disclosure behavior was
796 clarified. Finally, based on the hypothesis that shareholder composition influences the degree of voluntary disclosure
797 (Hypothesis 5 (H5)), the effect of shareholdings in other corporations on voluntary disclosure behavior was
798 demonstrated.
799 Fig 9 visualizes the impact of each Explanation variable on voluntary disclosure. Each Explanation variable in the
800 top and bottom rows is categorized according to whether it increases or inhibits the likelihood of voluntary disclosure,
801 with the up-right variable increasing the likelihood of disclosure and the down-right variable decreasing the
802 likelihood of disclosure.

803

804 Fig9. **Visualization of the Probability & Impact of Voluntary Disclosure Using Logistic Regression**
805 Visualizes the impact of each explanatory variable on voluntary disclosure using logistic regression. The variables are categorized as Upward, Downward,
806 or Other. Upward variables increase the likelihood of voluntary disclosure, while Downward variables decrease it. Other variables show little to no effect.
807 The length of each bar represents the probability of voluntary disclosure, and the Coefficient value next to each bar indicates the strength and direction
808 of the impact. Positive coefficients promote disclosure, while negative coefficients suppress it. The line at 0.5 marks the threshold above which disclosure
809 is more likely to occur.

810

811 This chart provides an intuitive overview of the factors that influence disclosure behavior and can serve as a guide
812 to which factors a company should focus on its disclosure strategy.

##### 813 In brief summary, the study yielded the following findings

814 1.	**Impact of Financial Indicators**
815 o	The larger the **size of the company** (total assets and sales), the more voluntary
816 disclosure tends to be promoted.
817 o	**Profitability** (e.g., operating margin, price-to-earnings ratio) has a complex effect on
818 voluntary disclosure. A trend toward restrained disclosure was identified, especially for Companies
819 with high PERs (price-to-earnings ratios).

##### 820 2. Impact of Company Characteristics

821 o	The number of **employees** and **ISO certifications** (e.g., ISO 14001 and ISO 45001)
822 are factors that actively promote voluntary disclosure.

| 823 |  | - On the other hand, **listing on a foreign market** acts as a factor that restrains disclosure |
| --- | --- | --- |
| 824 |  | behavior. |
| 825 | 3. | **Impact of Listed Markets** |
| 826  827 |  | - Companies listed on the prime market are more active in voluntary disclosure due to   the regulatory environment and transparency requirements from investors. |
| 828 |  | - Voluntary disclosure tends to be restrained for companies listed on the Standard, |
| 829 |  | Growth, and Local markets. |
| 830 | 4. | **Impact of Industry Classification** |
| 831 |  | - A trend toward active voluntary disclosure was observed in certain industries, such as |

832	manufacturing and information and telecommunications.

833	o	On the other hand, there is a marked tendency for disclosure to be suppressed in

834	agriculture, forestry, fisheries, mining, and local public service-related industries.

| 835 | 5. | **Impact of Shareholder Composition** |
| --- | --- | --- |
| 836 |  | - Voluntary disclosure is encouraged when a financial institution is a major shareholder. |
| 837 |  | - For companies with many other corporate shareholders, disclosure is restrained to |

838 protect their competitive advantage.

##### 839 6. Other Findings

840 o	**Regional characteristics**: companies listed only on local markets tend to be less active
841 in voluntary disclosure.
842 o	**Chronological change**: The content of disclosure is changing as companies move
843 toward integrated reporting and adopt new reporting formats.
844 o	**International comparison**: Compared to mandatory disclosure standards in other
845 countries, Japanese companies have chosen to pursue an autonomous disclosure strategy.
846 o
847 As an academic contribution, this study adds a new perspective on the impact of voluntary disclosure based on
848 industry classification and shareholder composition and reveals detailed industry differences that have not been
849 captured in previous studies. The study also delved into the previously unexplored impact of ISO certification and
850 shareholder composition on corporate disclosure behavior. Furthermore, the method of classifying Explanation
851 variables into "up," "down," and "other" shapes provides a new theoretical framework for voluntary disclosure
852 behavior.
853 As a practical contribution, the study provides suggestions for companies to formulate disclosure strategies more
854 effectively by understanding specifically which Explanation variables influence disclosure behavior. In particular,
855 taking into account the degree of influence of each variable shown in Fig 7 will enable companies to formulate
856 disclosure policies that are more in tune with the competitive environment and investor expectations. In addition, by
857 clarifying the impact of ISO certification and differences in shareholder composition on disclosure behavior, it is
858 possible to provide guidance on what strategies companies should adopt with respect to disclosure.
859 Additional research is needed to address the above new findings from future studies.
860 References
861 1.	POLOVYK Y, KOROL S (2024) Non-financial reporting in the industrial sector. SF 155 (3): 125–142.
862 2.	FOMETESCU A, HATEGAN CD (2024) The Non-Financial Information Contribution to Value Creation for

863	Companies. AUDITF 22 (173): 177–188.

864 3.	García-Meca E, Sánchez-Ballesta JP (2010) The Association of Board Independence and Ownership
865 Concentration with Voluntary Disclosure: A Meta-analysis. European Accounting Review 19 (3): 603–627.
866 4.	Carroll AB (1991) The Pyramid of Corporate Social Responsibility: Toward the Moral Management of
867 Organizational Stakeholders. Business Horizons 34 (4): 39–48.
868 5.	Schuster P OV (2006) The Trend toward Voluntary Corporate Disclosures. Management Accounting Quarterly
869 2006;7(2):1. Available: https://[www.proquest.com/scholarly-journals/trend-toward-voluntary-corporate-](http://www.proquest.com/scholarly-journals/trend-toward-voluntary-corporate-)
870 disclosures/docview/222799964/se-2?accountid=25225.
871 6.	Cynthia A. Williams, Williams CA (1999) The Securities and Exchange Commission and Corporate Social
872 Transparency. Harvard Law Review 112 (6): 1197.
873 7.	Bloomfield RJ (2012) A Pragmatic Approach to More Efficient Corporate Disclosure. Accounting Horizons

874	26 (2): 357–370.

875 8.	David Lenter & Joel Slemrod, Lenter D, Slemrod J, Shackelford D (2003) Public Disclosure of Corporate Tax
876 Return Information_Accounting, Economics, and Legal Perspectives // Public Disclosure of Corporate Tax
877 Return Information: Accounting, Economics, and Legal Perspectives. National Tax Journal 56 (4): 803–830.
878 9.	Lev B (1992) Information Disclosure Strategy. California Management Review 34 (4): 9–32.
879 10. Doan MA, McKie D (2017) Financial investigations: Auditing research accounts of communication in
880 business, investor relations, and public relations (1994–2016). Public Relations Review 43 (2): 306–313.
881 11. Velte P, Stawinoga M (2017) Integrated reporting: The current state of empirical research, limitations and
882 future research implications. J Manag Control 28 (3): 275–320.
883 12. Chauvey J-N, Giordano-Spring S, Cho CH, Patten DM (2015) The Normativity and Legitimacy of CSR
884 Disclosure: Evidence from France. J Bus Ethics 130 (4): 789–803.
885 13. Fiechter P, Hitz J-M, Lehmann N (2017) Real Effects of Disclosure Regulation: Evidence from the European
886 Union's CSR Directive. SSRN Electronic Journal.
887 14. Carnini Pulino S, Ciaburri M, Magnanelli BS, Nasta L (2022) Does ESG Disclosure Influence Firm
888 Performance. Sustainability 14 (13): 7595.
889 15. García-Sánchez I-M, Noguera-Gámez L (2017) Integrated information and the cost of capital. International
890 Business Review 26 (5): 959–975.
891 16. Hossain M, Reaz M (2007) The determinants and characteristics of voluntary disclosure by Indian banking
892 companies. Corp Soc Responsibility Env 14 (5): 274–288.
893 17. Szewieczek A, Dratwińska-Kania B, Ferens A (2021) Business Model Disclosure in the Reporting of Public
894 Companies—An Empirical Study. Sustainability 13 (18): 10088.
895 18. Heydarnasab, D., & Rezaei, F. (2016) the-relationship-between-the-financial-ratios-and-information-
896 disclosure-level-in-companies. Available: https://civilica.com/doc/437071/.
897 19. Dedman E, Lennox C (2009) Perceived competition, profitability and the withholding of information about
898 sales and the cost of sales. Journal of Accounting and Economics 48 (2-3): 210–230.
899 20. Brockman P, Unlu E (2011) Earned/contributed capital, dividend policy, and disclosure quality: An
900 international study. Journal of Banking & Finance 35 (7): 1610–1625.
901 21. Ni X, Zhang H (2019) Mandatory corporate social responsibility disclosure and dividend payouts: evidence
902 from a quasi‐natural experiment. Accounting & Finance 58 (5): 1581–1612.
903 22. Bulut Sürdü F, Çalışkan AÖ, Esen E (2020) Human Resource Disclosures in Corporate Annual Reports of
904 Insurance Companies: A Case of Developing Country. Sustainability 12 (8): 3452.
905 23. ADAMS, C. A. ET AL., ADAMS CA, HILL W-Y, Roberts CB (1998) CORPORATE SOCIAL REPORTING
906 PRACTICES IN WESTERN EUROPE: LEGITIMATING CORPORATE BEHAVIOUR. The British
907 Accounting Review 30 (1): 1–21.
908 24. Hossain M, Reaz M (2007) The determinants and characteristics of voluntary disclosure by Indian banking
909 companies. Corp Soc Responsibility Env 14 (5): 274–288.
910 25. Okoye, Emma Ik., Adeniyi, Segun Idowu (2018) Company age and voluntary corporate social disclosure in
911 Nigeria : a study of selected listed manufacturing firms on the Nigerian stock exchange. Academic journal of
912 economic studies. Available: [http://hdl.handle.net/11159/2190.](http://hdl.handle.net/11159/2190)
913 26. Nanda AP, Nahumury J (2019) The effect of financial performance, board of commissioners, blockholder
914 ownership, auditor type and firm age on voluntary disclosure. TIAR 8 (2): 145–161.
915 27. Rahmi Oktriani, Fefri Indra Arza Pengaruh Listing Age dan Ownership Dispersion Terhadap Luas
916 Pengungkapan Sukareka dengan Firm Size Sebagai Variabel Pemoderasi (Studi Empiris pada Perusahaan
917 Manufaktur yang Terdaftar di BEI tahun 2014-2016).
918 28. Elkins H, Entwistle G (2018) A commentary on accounting standards and the disclosure problem: Exploring a
919 way forward. Journal of International Accounting, Auditing and Taxation 33: 79–89.
920 29. Iatridis G (2008) Accounting disclosure and firms' financial attributes: Evidence from the UK stock market.
921 International Review of Financial Analysis 17 (2): 219–241.
922 30. C. Sherman Cheung, Jason Lee, Sherman Cheung C, Lee J (1995) Disclosure environment and listing on
923 foreign stock exchanges. Journal of Banking & Finance 19 (2): 347–362.
924 31. Yu EP, van Luu B (2021) International variations in ESG disclosure – Do cross-listed companies care more.
925 International Review of Financial Analysis 75: 101731.
926 32. Carol A. Frost and Grace Pownall, Frost CA, Pownall G (1994) Accounting Disclosure Practices in the United
927 States and the United Kingdom. Journal of Accounting Research, Vol. 32, No. 1 // 32 (1): 75–102.
928 33. Shi Y, Magnan M, Kim J-B (2012) Do countries matter for voluntary disclosure? Evidence from cross-listed
929 firms in the US. J Int Bus Stud 43 (2): 143–165.
930 34. Cheng Q, LO KI (2006) Insider Trading and Voluntary Disclosures. J of Accounting Research 44 (5): 815–

931	848.

932 35. Kumar P, Langberg N, Oded J, Sivaramakrishnan K (2017) Voluntary disclosure and strategic stock
933 repurchases. Journal of Accounting and Economics 63 (2-3): 207–230.
934 36. Chariri A, Januarti I, Yuyetta ENA (2023) ISO Certification, Firm Characteristics and Carbon Emission
935 Disclosure. IOP Conf. Ser.: Earth Environ. Sci. 1248 (1): 12024.
936 37. Harlinda Siska Pradini, Endang Kiswara1 The Analysis of Information Content towards Greenhouse Gas
937 Emissions Disclosure In Indonesia’s Companies. Available:
938 https://ejournal3.undip.ac.id/index.php/accounting/article/view/3317.
939 38. Adhikari A, Tondkar RH (1992) Environmental Factors Influencing Accounting Disclosure Requirements of
940 Global Stock Exchanges. Financ Manag Account 4 (2): 75–105.
941 39. Lardon A, Deloof M (2014) Financial disclosure by SMEs listed on a semi-regulated market: evidence from
942 the Euronext Free Market. Small Bus Econ 42 (2): 361–385.
943 40. Suijs J, Wielhouwer JL (2019) Disclosure policy choices under regulatory threat. The RAND J of Economics

944	50 (1): 3–28.

945 41. Gary K. Meek, Clare B. Roberts and Sidney J. Gray, Meek GK, Roberts CB, Gray SJ (1995) Factors
946 Influencing Voluntary Annual Report Disclosures by U.S., U.K. and Continental European Multinational
947 Corporations // Factors Influencing Voluntary Annual Report Disclosures By U.S., U.K. and Continental
948 European Multinational Corporations. J Int Bus Stud 26 (3): 555–572.
949 42. Broberg P, Tagesson T, Collin S-O (2010) What explains variation in voluntary disclosure? A study of the
950 annual reports of corporations listed on the Stockholm Stock Exchange. J Manag Gov 14 (4): 351–377.
951 43. Bewley K, Li Y, editors (2000) Disclosure of environmental information by Canadian manufacturing
952 companies: A voluntary disclosure perspective. Bingley: Emerald (MCB UP ).
953 44. Rouf, Abdur M, Al-Harun, Abdullah Ownership structure and voluntary disclosure in annual reports of
954 Bangladesh. Available: https://[www.econstor.eu/handle/10419/188019.](http://www.econstor.eu/handle/10419/188019)
955 45. Patelli L, Prencipe A (2007) The Relationship between Voluntary Disclosure and Independent Directors in the
956 Presence of a Dominant Shareholder. European Accounting Review 16 (1): 5–33.
957 46. Bae SM, Masud MAK, Kim JD (2018) A Cross-Country Investigation of Corporate Governance and
958 Corporate Sustainability Disclosure: A Signaling Theory Perspective. Sustainability 10 (8): 2611.
959 47. Flower J (2015) The International Integrated Reporting Council: A story of failure. Critical Perspectives on
960 Accounting 27: 1–17.

[Click here to access/download;Figure;Fig_1.tif](https://www2.cloud.editorialmanager.com/pone/download.aspx?id=38827712&guid=58c6185c-e494-4f0a-b321-c364f0e29ba4&scheme=1)[<https://www2.cloud.editorialmanager.com/pone/download.aspx?id=38827712&guid=58c6185c-e494-4f0a-b321-c364f0e29ba4&scheme=1>](https://www2.cloud.editorialmanager.com/pone/download.aspx?id=38827712&guid=58c6185c-e494-4f0a-b321-c364f0e29ba4&scheme=1)

[Click here to access/download;Figure;Fig_2.tif](https://www2.cloud.editorialmanager.com/pone/download.aspx?id=38827713&guid=9191fd8e-d813-4f04-8774-1fe040a1eb09&scheme=1)[<https://www2.cloud.editorialmanager.com/pone/download.aspx?id=38827713&guid=9191fd8e-d813-4f04-8774-1fe040a1eb09&scheme=1>](https://www2.cloud.editorialmanager.com/pone/download.aspx?id=38827713&guid=9191fd8e-d813-4f04-8774-1fe040a1eb09&scheme=1)

[Click here to access/download;Figure;Fig_3.tif](https://www2.cloud.editorialmanager.com/pone/download.aspx?id=38827714&guid=6a32f5a5-d6c0-4262-9a70-7733cca1bffa&scheme=1)[<https://www2.cloud.editorialmanager.com/pone/download.aspx?id=38827714&guid=6a32f5a5-d6c0-4262-9a70-7733cca1bffa&scheme=1>](https://www2.cloud.editorialmanager.com/pone/download.aspx?id=38827714&guid=6a32f5a5-d6c0-4262-9a70-7733cca1bffa&scheme=1)

[Click here to access/download;Figure;Fig_4.tif](https://www2.cloud.editorialmanager.com/pone/download.aspx?id=38827715&guid=7971a5ef-3725-4df3-a10c-b2cb22c1d56f&scheme=1)[<https://www2.cloud.editorialmanager.com/pone/download.aspx?id=38827715&guid=7971a5ef-3725-4df3-a10c-b2cb22c1d56f&scheme=1>](https://www2.cloud.editorialmanager.com/pone/download.aspx?id=38827715&guid=7971a5ef-3725-4df3-a10c-b2cb22c1d56f&scheme=1)

[Click here to access/download;Figure;Fig_5.tif](https://www2.cloud.editorialmanager.com/pone/download.aspx?id=38827716&guid=9195873a-841b-4aef-a8f5-27aafb6ed277&scheme=1)[<https://www2.cloud.editorialmanager.com/pone/download.aspx?id=38827716&guid=9195873a-841b-4aef-a8f5-27aafb6ed277&scheme=1>](https://www2.cloud.editorialmanager.com/pone/download.aspx?id=38827716&guid=9195873a-841b-4aef-a8f5-27aafb6ed277&scheme=1)

[Click here to access/download;Figure;Fig_6.tif](https://www2.cloud.editorialmanager.com/pone/download.aspx?id=38827717&guid=71b407f0-542d-4a4c-9579-f9cbe25d27f1&scheme=1)[<https://www2.cloud.editorialmanager.com/pone/download.aspx?id=38827717&guid=71b407f0-542d-4a4c-9579-f9cbe25d27f1&scheme=1>](https://www2.cloud.editorialmanager.com/pone/download.aspx?id=38827717&guid=71b407f0-542d-4a4c-9579-f9cbe25d27f1&scheme=1)

[Click here to access/download;Figure;Fig_7.tif](https://www2.cloud.editorialmanager.com/pone/download.aspx?id=38827718&guid=6417da07-d078-4981-960b-af2930c9c8aa&scheme=1)[<https://www2.cloud.editorialmanager.com/pone/download.aspx?id=38827718&guid=6417da07-d078-4981-960b-af2930c9c8aa&scheme=1>](https://www2.cloud.editorialmanager.com/pone/download.aspx?id=38827718&guid=6417da07-d078-4981-960b-af2930c9c8aa&scheme=1)

[Click here to access/download;Figure;Fig_8-1.tif](https://www2.cloud.editorialmanager.com/pone/download.aspx?id=38827719&guid=2a33eebe-40b8-4db5-b76d-e14cac314f41&scheme=1)[<https://www2.cloud.editorialmanager.com/pone/download.aspx?id=38827719&guid=2a33eebe-40b8-4db5-b76d-e14cac314f41&scheme=1>](https://www2.cloud.editorialmanager.com/pone/download.aspx?id=38827719&guid=2a33eebe-40b8-4db5-b76d-e14cac314f41&scheme=1)

[Click here to access/download;Figure;Fig_8-2.tif](https://www2.cloud.editorialmanager.com/pone/download.aspx?id=38827720&guid=e3bcf4ef-2ae0-409c-bd69-a6749c35aa66&scheme=1)[<https://www2.cloud.editorialmanager.com/pone/download.aspx?id=38827720&guid=e3bcf4ef-2ae0-409c-bd69-a6749c35aa66&scheme=1>](https://www2.cloud.editorialmanager.com/pone/download.aspx?id=38827720&guid=e3bcf4ef-2ae0-409c-bd69-a6749c35aa66&scheme=1)

[Click here to access/download;Figure;Fig_8-3.tif](https://www2.cloud.editorialmanager.com/pone/download.aspx?id=38827722&guid=0e079df5-76e0-4909-8851-1f0ec48512df&scheme=1)[<https://www2.cloud.editorialmanager.com/pone/download.aspx?id=38827722&guid=0e079df5-76e0-4909-8851-1f0ec48512df&scheme=1>](https://www2.cloud.editorialmanager.com/pone/download.aspx?id=38827722&guid=0e079df5-76e0-4909-8851-1f0ec48512df&scheme=1)

[Click here to access/download;Figure;Fig_8-4.tif](https://www2.cloud.editorialmanager.com/pone/download.aspx?id=38827723&guid=b61900b2-d9e9-4ef8-a16b-548527b65906&scheme=1)[<https://www2.cloud.editorialmanager.com/pone/download.aspx?id=38827723&guid=b61900b2-d9e9-4ef8-a16b-548527b65906&scheme=1>](https://www2.cloud.editorialmanager.com/pone/download.aspx?id=38827723&guid=b61900b2-d9e9-4ef8-a16b-548527b65906&scheme=1)

[Click here to access/download;Figure;Fig_8-5.tif](https://www2.cloud.editorialmanager.com/pone/download.aspx?id=38827724&guid=85a80545-0a75-4703-9ae4-392bc80c204d&scheme=1)[<https://www2.cloud.editorialmanager.com/pone/download.aspx?id=38827724&guid=85a80545-0a75-4703-9ae4-392bc80c204d&scheme=1>](https://www2.cloud.editorialmanager.com/pone/download.aspx?id=38827724&guid=85a80545-0a75-4703-9ae4-392bc80c204d&scheme=1)

[Click here to access/download;Figure;Fig_8-6.tif](https://www2.cloud.editorialmanager.com/pone/download.aspx?id=38827725&guid=001c29e2-890a-4782-8e5b-c83b3a0f18ac&scheme=1)[<https://www2.cloud.editorialmanager.com/pone/download.aspx?id=38827725&guid=001c29e2-890a-4782-8e5b-c83b3a0f18ac&scheme=1>](https://www2.cloud.editorialmanager.com/pone/download.aspx?id=38827725&guid=001c29e2-890a-4782-8e5b-c83b3a0f18ac&scheme=1)

[Click here to access/download;Figure;Fig_9.tif](https://www2.cloud.editorialmanager.com/pone/download.aspx?id=38827726&guid=329e34bc-099c-4f96-b177-3ad5f8a0dfa7&scheme=1)[<https://www2.cloud.editorialmanager.com/pone/download.aspx?id=38827726&guid=329e34bc-099c-4f96-b177-3ad5f8a0dfa7&scheme=1>](https://www2.cloud.editorialmanager.com/pone/download.aspx?id=38827726&guid=329e34bc-099c-4f96-b177-3ad5f8a0dfa7&scheme=1)

[Click here to access/download](https://www2.cloud.editorialmanager.com/pone/download.aspx?id=38827727&guid=77a9ce4c-a712-414f-865e-c51050d8fdbb&scheme=1)

[**Supporting Information** S1.csv](https://www2.cloud.editorialmanager.com/pone/download.aspx?id=38827727&guid=77a9ce4c-a712-414f-865e-c51050d8fdbb&scheme=1)

[Click here to access/download](https://www2.cloud.editorialmanager.com/pone/download.aspx?id=38827728&guid=252c5bda-6909-4de5-bfb2-71c9e3e19a0d&scheme=1)

[**Supporting Information** S2.xlsx](https://www2.cloud.editorialmanager.com/pone/download.aspx?id=38827728&guid=252c5bda-6909-4de5-bfb2-71c9e3e19a0d&scheme=1)

[Click here to access/download](https://www2.cloud.editorialmanager.com/pone/download.aspx?id=38827729&guid=85330393-16ba-4a39-8c5b-6eb8052135cb&scheme=1)

[**Supporting Information** S3.docx](https://www2.cloud.editorialmanager.com/pone/download.aspx?id=38827729&guid=85330393-16ba-4a39-8c5b-6eb8052135cb&scheme=1)

[Click here to access/download](https://www2.cloud.editorialmanager.com/pone/download.aspx?id=38827730&guid=73a71dfd-907d-4990-8095-bfe1fb1f794d&scheme=1)

[**Supporting Information** S4.xlsx](https://www2.cloud.editorialmanager.com/pone/download.aspx?id=38827730&guid=73a71dfd-907d-4990-8095-bfe1fb1f794d&scheme=1)

[Click here to access/download](https://www2.cloud.editorialmanager.com/pone/download.aspx?id=38827731&guid=faeee942-fbed-4d23-bf93-064aed8cd785&scheme=1)

[**Supporting Information** S5.xlsx](https://www2.cloud.editorialmanager.com/pone/download.aspx?id=38827731&guid=faeee942-fbed-4d23-bf93-064aed8cd785&scheme=1)
