# Supplementary material for: Determinants of voluntary disclosure: An empirical analysis of financial, market, and organizational factors
Source: PLoS One. 2025 Jun 4;20(6):e0324625. doi: 10.1371/journal.pone.0324625 (PMC12136342; doi:10.1371/journal.pone.0324625)
Supplement: Attachment — Submitted filename: PONE-D-25-00849_Response Letter_ver2.2_2025_0321_0608.docx [file pone.0324625.s011.docx]

Dear Editor,

We sincerely thank you and the reviewers for your valuable feedback on our manuscript titled "Determinants of Voluntary Disclosure: An Empirical Analysis of Financial, Market, and Organizational Factors" (PONE-D-25-00849). We have carefully addressed all comments provided by Reviewer 1 and Reviewer 2. Below, we summarize our revisions and clarify the current status of the manuscript.

**Summary of Revisions**

We have carefully revised our manuscript in response to the reviewers' comments. Specifically:

- Abstract: Revised according to the suggested 3P+1T structure and limited to approximately 200 words.
- Hypotheses: Clarified theoretical foundations by explicitly referencing existing literature from our initial literature review (Section 2.1.1), without adding new references that could potentially disrupt our established theoretical framework.
- Technical term definitions: Provided clear and explicit definitions of terms such as CSR, ESG, ISO certifications, PER, and IFRS.
- Formatting and references: Standardized formatting and citation style to fully comply with the submission guidelines of PLOS ONE.
- Figures and tables: Strengthened cross-referencing within the manuscript to enhance readability, despite figures and tables being positioned at the end as per journal submission guidelines.

Regarding Reviewer 1’s comment about providing supporting literature for each hypothesis, we carefully considered adding new references. However, our supervisor advised that introducing new literature might inadvertently disrupt the logical coherence of the original manuscript. Therefore, we decided to strengthen each hypothesis discussion by explicitly referencing relevant studies already reviewed in the literature review section (section 2.1.1), rather than adding new references. This approach ensures consistency and avoids unnecessary complexity.

We believe these modifications comprehensively address the reviewers' feedback and significantly enhance the manuscript’s clarity and coherence. Therefore, we respectfully request that the revised manuscript be considered without additional major revisions.

Thank you again for your valuable feedback and kind consideration.

Sincerely,

Yuichiro Nakai and Mitsuo Yoshida

University of Tsukuba, Tokyo, Japan

**Response to Reviewer 1**

**Major Issues:**

**1. Abstract Revision**

- **Reviewer Comment:** "This abstract is engaging! However, consider reorganizing it using the 3P+1T structure: Purpose, Procedure (Methods), Primary Findings, and Theory. Try to reduce it to 200 words."
- **Response:** Thank you for the valuable suggestion. We have revised the abstract following the 3P+1T structure and ensured that it does not exceed 200 words. The revised abstract now clearly outlines the research purpose, methodology, key findings, and theoretical contribution. *(See revised manuscript, Abstract section: page 1.)*
- **Revised Abstract**
  This study systematically examines the determinants of voluntary disclosure by listed companies in Japan, where non-financial disclosure is not legally mandated. Using quarterly data from 5,915 companies between 2009 and 2024, a logistic regression analysis was conducted to assess the influence of financial indicators, company characteristics, stock exchange, industry classification, and shareholder composition.　The results reveal that ISO certification (ISO 14001 and ISO 45001) significantly promotes voluntary disclosure. Firm size and listing on a prime market are positively associated with disclosure, whereas foreign market listings tend to suppress disclosure—contrary to conventional theories. Additionally, manufacturing and IT sectors are proactive in disclosure, whereas the agriculture, forestry, fishing, and mining sectors are more reluctant. Shareholder composition also plays a crucial role, with financial institution shareholders promoting disclosure and corporate shareholders suppressing it.　By leveraging Japan’s unique voluntary disclosure environment, this study offers new insights into corporate transparency and the strategic design of disclosure policies.

**2. Strengthening Hypothesis Discussion with Literature Support**

- **Reviewer Comment:** "For each hypothesis discussion, please provide supporting literature to strengthen your analysis."
- **Response:** We thank the reviewer for this insightful comment. We have clarified the theoretical foundation of each hypothesis using the literature already reviewed and included in our manuscript. To maintain clarity and avoid potential confusion from adding new sources, we refrained from incorporating additional references. We believe the existing literature sufficiently supports our hypotheses. (See revised manuscript, Literature Review and Hypotheses sections, pages 4–7.)
- ***Specific changes added at the end of each hypothesis:***

***H1:***

- These arguments are supported by prior research emphasizing that voluntary disclosure, from an agency theory perspective, reduces information asymmetry and enhances transparency, thereby building trust with investors and regulators [6]. Conversely, excessive disclosure can pose competitive risks, requiring companies to strategically balance transparency and competitive advantage [7,8].

***H2:***

- These arguments are supported by previous research indicating that corporate disclosure is strategically influenced by the company's environment and market expectations [9], and that voluntary disclosures, especially integrated reports including sustainability and governance information, enhance decision usefulness and stakeholder relationship management [11].

***H3:***

- This hypothesis is grounded in previous studies suggesting that companies strategically decide on disclosure depending on their competitive environment and market expectations [9], and that companies enhance transparency through voluntary disclosures to increase investor trust and meet market expectations [10].

***H4:***

- This hypothesis draws upon earlier research indicating that companies strategically determine their disclosure behavior in response to specific regulatory pressures and competitive environments, and that industry-specific characteristics critically shape corporate voluntary disclosure strategies [7,9].

***H5:***

- This hypothesis is supported by prior research indicating that corporate disclosure strategies are significantly influenced by their competitive environment and strategic considerations related to maintaining competitive advantages, which vary according to shareholder composition and corporate governance structures [7,9,10] .

**Minor Issues:**

**3. Clarification of Explanatory Variable (XXX)**

- **Reviewer Comment:** "In line 386, what is the explanatory variable XXX? Should the authors provide further clarification, or is this a typo?"
- **Response:** We appreciate the reviewer pointing this out. All variable definitions and explanations are provided in Supporting Information 2 (S2: Explanation Variables).

**4. Formatting and Reference Consistency**

- **Reviewer Comment:** "The article’s format and references appear inconsistent. Please double-check the formatting."
- **Response:** Thank you for bringing this to our attention. We have thoroughly reviewed the manuscript to ensure consistency in formatting, reference citation style, and bibliography structure. Any inconsistencies in formatting have been corrected to align with the journal’s submission guidelines. *Specifically, we ensured PLOS ONE uniform citation style, consistent spacing, alignment, and font usage throughout the manuscript. (See revised manuscript, entire document.)*

**Response to Reviewer 2**

**Overall Reviewer comment**

Dear Author, first of all thank you for your article.

The purpose of your paper is clear, but the outputs and conclusions of the analysis are very confusing due to the way it is presented and the study design. This makes the article difficult to understand.

Similarly, the data and figures in your study are too discrete, making it difficult to analyze the paper as a whole and therefore not easily understood. There are also some corrections in the attached file

It would be useful to revise the paper again and make it easier to read and understand with minor revisions.

Thank you

**Our Response:**
We appreciate the reviewer’s constructive feedback and fully acknowledge the concern regarding readability and clarity.

We have carefully reviewed your valuable comments and further revised our manuscript accordingly.
Specifically, we have clarified references to all figures in the manuscript text, provided explicit definitions for technical terms, and standardized all formatting and references to align strictly with journal guidelines. We believe these modifications fully address all concerns raised, significantly enhancing the manuscript’s readability, clarity, and overall coherence.

We recognize that the positioning of figures and tables at the end of the manuscript, as required by the submission guidelines of PLOS ONE (https://journals.plos.org/plosone/s/submission-guidelines#loc-figures-and-tables), may contribute to the difficulty in following the paper.

To address your concerns, we have implemented the following measures to enhance the readability and clarity of our manuscript:

**Comment1**

- 18th Line Comment ("CSR reports and ESG reports.")
- **Reviewer Comment**: "CSR and ESG abbreviations should be written clearly first, and the terms should be explained."
- **Response:** We appreciate the reviewer pointing this out. Yes, we will clarify these terms clearly at their first occurrence.
- **Revised version:** "Corporate Social Responsibility (CSR) reports and Environmental, Social, and Governance (ESG) reports."

**Comment2**

- Lines 78–82 Comment
- **Reviewer Comment**: "P/E ratios, ISO 14001 and ISO 45001 terms should be explained."
- **Response:** We appreciate the reviewer pointing this out. Yes, we will provide additional explanations for these terms.
- **Revised version:** "Companies with higher Price-to-Earnings (P/E) ratios, indicating market expectations for growth, are less likely to voluntarily disclose due to competitive reasons. ISO certifications, such as ISO 14001 (environmental management systems) and ISO 45001 (occupational health and safety management systems), actively promote voluntary disclosure."

**Comment3**

- 141st Line Comment ("and the IFRS Foundation")
- **Reviewer Comment**: "IFRS Foundation should be explained."
- **Response:** We appreciate the reviewer pointing this out. Yes, we will provide an explanation.
- **Revised version:** "and the International Financial Reporting Standards (IFRS) Foundation, which develops global accounting standards."

**Comment4**

- Lines 151–153 Comment (Fig. 1)
- **Reviewer Comment**: " In the article, all figures are given at the end, but this makes it difficult to understand the paper. It would be useful to explain the figures in their place."
- **Response:** We agree that placing figures close to their mention improves readability. However, PLOS ONE submission guidelines require figures to be placed after the manuscript text. Upon publication, figures will be positioned appropriately within the text.

**Comment5**

- 371st Line Comment
- **Reviewer Comment**: "What do the highlighted numbers mean?"
- **Response:** These numbers represent the count of explanatory variables. We have added more clarifying information.
- **Revised version:**

H1: Nine items of β1_Financial Indicators (β1-1 to β1-9)

H2: Eight items of β2_Company Characteristics (β2-1 to β2-8)

H3: Four items of β3_Market-Related Factors (β3-1 to β3-4)

H4: Thirty-three items of β4_Industry Classification (β4-1 to β4-33)

H5: Five items of β5_Shareholding Ratios (β5-1 to β5-5)

Point in time information (quarterly variable: β6_ Quarter)

**Comment6**

- Lines 383–384 Comment
- **Reviewer Comment**: "None of the variables in the equation has an explanation. What is their meaning?"
- **Response:** All variable definitions and explanations are provided in Supporting Information 2 (S2: Explanation Variables).
- **Revised version:**

In addition, the Explanation variable (See S2: Explanation Variables) falls into the five categories described above.

**Comment7**

- 386th Line Comment (XXX)
- **Reviewer Comment**: "XXX? What do you mean by that?"
- **Response:** We initially intended to refer to Supporting Information 2 (S2: Explanation Variables).
- **Revised version:**

In addition, the Explanation variable (See S2: Explanation Variables) falls into the five categories described above.

**Comment8**

- 787th Line Comment ("Conclusion")
- **Reviewer Comment**: " Because the data and figures are so discrete, the article as a whole is difficult to analyze and therefore not easily understood.　The purpose of the study is clear, but the outputs and results of the analysis are very confusing due to the way it is presented and the study design."

**Response:** We appreciate the reviewer’s constructive feedback and fully acknowledge the concern regarding readability and clarity. 　We have carefully reviewed your valuable comments and further revised our manuscript accordingly.
Specifically, we have clarified references to all figures in the manuscript text, provided explicit definitions for technical terms, and standardized all formatting and references to align strictly with journal guidelines. We believe these modifications fully address all concerns raised, significantly enhancing the manuscript’s readability, clarity, and overall coherence. We recognize that the positioning of figures and tables at the end of the manuscript, as required by the submission guidelines of PLOS ONE(https://journals.plos.org/plosone/s/submission-guidelines#loc-figures-and-tables), may contribute to the difficulty in following the paper.
